# An update on extra-oral bitter taste receptors

**DOI:** 10.1186/s12967-021-03067-y

**Published:** 2021-10-21

**Authors:** Tuzim Kamila, Korolczuk Agnieszka

**Affiliations:** Department of Clinical Pathomorphology, Medical University of Lublin, ul. Jaczewskiego 8b, 20-090 Lublin, Poland

**Keywords:** G-protein coupled receptors (GPCRs), Bitter taste-sensing type 2 receptors (TAS2Rs), TAS2R polymorphisms, Bitter compounds, Extraoral tissues, Innate immunity, Muscle contractility, Cancer

## Abstract

Bitter taste-sensing type 2 receptors (TAS2Rs or T2Rs), belonging to the subgroup of family A G-protein coupled receptors (GPCRs), are of crucial importance in the perception of bitterness. Although in the first instance, TAS2Rs were considered to be exclusively distributed in the apical microvilli of taste bud cells, numerous studies have detected these sensory receptor proteins in several extra-oral tissues, such as in pancreatic or ovarian tissues, as well as in their corresponding malignancies. Critical points of extra-oral TAS2Rs biology, such as their structure, roles, signaling transduction pathways, extensive mutational polymorphism, and molecular evolution, have been currently broadly studied. The TAS2R cascade, for instance, has been recently considered to be a pivotal modulator of a number of (patho)physiological processes, including adipogenesis or carcinogenesis. The latest advances in taste receptor biology further raise the possibility of utilizing TAS2Rs as a therapeutic target or as an informative index to predict treatment responses in various disorders. Thus, the focus of this review is to provide an update on the expression and molecular basis of TAS2Rs functions in distinct extra-oral tissues in health and disease. We shall also discuss the therapeutic potential of novel TAS2Rs targets, which are appealing due to their ligand selectivity, expression pattern, or pharmacological profiles.

## Background

Bitterness perception is considered a key defense mechanism against poisoning with potentially toxic substances. Although so far, direct relationship between bitterness and food toxicity has not been established [1], it is not worthy that many common bitterants, such as strychnine and nicotine, are toxic at low or high concentrations, or even fatal [2, 3]. Bitter taste is perceived via bitter taste-sensing type 2 receptors (TAS2Rs or T2Rs), the second largest group (25 members) of chemosensory G-protein coupled receptors (GPCRs) [4–7]. These are embedded not only in the plasma membrane of the type II taste receptor cells (TRCs) located in the taste buds of the tongue, soft palate, throat, and larynx [8], but also in several extra-oral tissues throughout the body. After Höfer’s group identified α-gustducin (bitter taste signalling molecule) in the intestinal epithelium in 1996 [9], the expression of TAS2Rs alone in the gastrointestinal (GI) and nasal cavity cells was confirmed in 2002 by Wu et al. [10] and in 2003 by Finger et al. [11]. Thus, the network of extra-oral TAS2Rs participate in various (patho)physiological processes in auto-, para-, and endocrine manners, which may differ from the perception of taste stimuli [12]. In the last decade, several studies have investigated the extra chemoreceptive roles of TAS2Rs in selected tissues and organs of the body in vitro and in vivo, as well as their possible clinical implications [12, 13]. Nevertheless, owing to the dynamic progress in research regarding these receptor proteins, a comprehensive review summarising these findings is required, which is the purpose of this article.

## The nature of the bitter taste receptors

### Genomic feature, protein structure and distribution of TAS2Rs

Twenty-five different TAS2Rs have been identified in humans (hTAS2Rs), 35 in mice (mTAS2Rs), 12 in cats (fTAS2Rs), and 3 different subtypes in chickens (ggTAS2Rs) [14[. As GPCRs, these proteins comprise seven α-helical transmembrane domains (TM1–TM7), an extracellular N-terminus with three extracellular loops (ECL1–ECL3), and an intracellular C-terminus with three intracellular loops (ICL1–ICL3) [15]. Genes encoding TAS2Rs are clustered on the short arm of chromosome 5 (5p15) and 12 (12p13), and on the long arm of chromosome 7 (7q31-7q35) [16]. They are selectively expressed in TRCs that do not express receptors for sweet and umami taste [17, 18]. Approximately 4–11 genes for TAS2Rs are co-expressed in one cell [19].

### A panel of bitterants

**Fig. 1:**
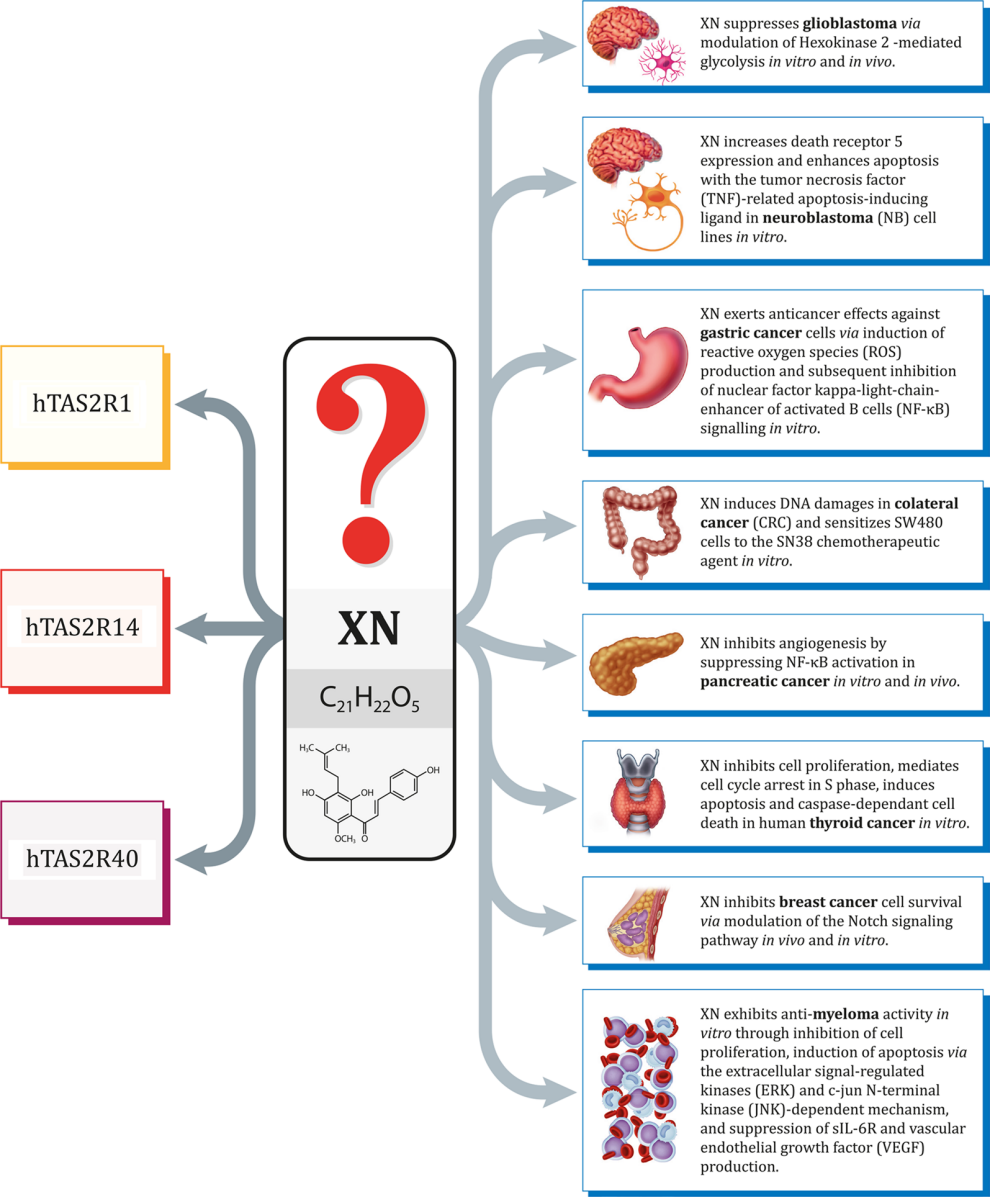
Xanthohumol (XN), a prenylated flavonoid isolated from Hops (*Humulus lupulus L.*) is an agonist of TAS2R1, 14, and 40. Simultaneously, it acts as a potent anticancer agent for multiple types of cancer. However, so far there are no evidence supporting a linkage between TAS2Rs and XN-mediated anti-tumor activity

In 2019, 1041 compounds were registered in the database of bitter compounds (BitterDB, http://bitterdb.agri.huji.ac.il) (a two-fold increase in the number of molecules compared to that in the original BitterDB developed in 2012), which included 51 peptides with confirmed bitter properties for humans or activating at least one TAS2R in one of four species such as human, chicken, cat, or mouse. In total, 306 molecules of natural origin and 58 synthetic compounds were isolated from the above group, while the remaining 677 ligands were not assigned to any of the above categories [14, 20]. Numerous bitter phytochemicals and their derivatives have been previously identified as promising options for improving the treatment of various diseases, including neoplastic disorders. Among them, for example, is xanthohumol [14] with well-described anti-cancer properties [21–28]. However, whether these anti-tumor activities are TAS2R-dependant at all is still unknown (Fig. 1).

Another recently pinpointed stimulating ligands for TAS2Rs are quorum sensing (QSM) molecules derived from both Gram-negative (such as acyl-homoserine lactones (AHLs), quinolones) [29, 30] and Gram-positive (for example competence stimulating peptides (CSPs)) [31–35] bacteria, as well as their metabolites (that is acetone, 2-butanone, 2-pentanone, 2-methylpropanal, dimethyl disulfide, methylmercaptan, γ-butyrolactone) [36]. CSP-1, for instance, acting as hTAS2R14 agonist, induced calcium signaling and secretion of cytokines CXCL-8/IL-8, tumour necrosis factor α (TNF-α) and IL-6 in gingival epithelial cells [31]. This strongly supports a role of TAS2Rs in modulating innate immune response and mediating interkingdom signaling through mammalian cell detection of bacterial products. Interestingly, many commonly used drugs are bitter, and therefore, they are likely to activate selected TAS2Rs. These include antibiotics (chloramphenicol, erythromycin, and ofloxacin), anti-malarial drugs (quinine [Q] and chloroquine [ChQ]), analgesics (acetamonifen), or thyrostatic agents (metimazole and propylthiouracil). Thus, bitter medications, in addition to the expected mechanism of action, can regulate several other, possibly undesirable, (patho)physiological processes via TAS2Rs [37].

o The 25 hTAS2Rs have varying receptive range with the number of known agonists extending from 151 for hTAS2R14 to zero for 5 orphan receptors (hTAS2R41,-42,-45,-48 and -60) [14, 38, 39]. Conversely, only 60% of the mTAS2Rs respond to known agonists (vs. 84% of the hTAS2Rs) [38]. It is also shown that some of the receptors, such as hTAS2R5, 8, 13, and 49, have more synthetic ligands identified, while others, such as hTAS2R1, 4, 7, 39, 40, 43, 44, and 47, present more natural agonists [38–40]. In turn, the example of dual affinity for both natural and synthetic ligands is hTAS2R38, which on the one hand interacts with such phytochemicals like limonin or sinigrin, and on the other with synthetics including chlorpheniramine or methimazole [38]. Similar to olfactory receptors, about 50% bitterants stimulate only one hTAS2R (such as noscapine, amygdalin, procainamide, and pirenzepine), while the other half activates from 2 to 9 or even 15 receptors, which mostly depends on the concentration of the reactant (for example, diphenidol, quinine, chlorphenamine, and sucralose) [14, 39].

### TAS2Rs polymorphisms

The human hTAS2Rs contain numerous single nucleotide polymorphisms (SNPs), which result in individual differences in perception and responsiveness to a bitter agonist. A classic example is that of hTAS2R38, the three most common polymorphisms (rs714598, rs1726866 and rs10246939) of which dictate the phenotypic diversity of taste sensitivity to phenylthiocarbamide (PTC), 6-n-propylthioutacyl (6-PTU), and chemically similar thiourea (N–C=S) moiety-containing chemicals. The differences are observed at amino acids at positions 49, 262, and 296. The above polymorphisms tend to co-segregate, resulting in two predominant haplotypes. The functional (“tasting”) allele encodes proline, alanine, and valine in the above positions (Pro49, Ala262, and Val295; henceforth the associated genotype is named PAV), while the non-functional allele encodes alanine, valine, and isoleucine (Ala49, Val 262, and Ile 296; the genotype is named AVI). People homozygous for the functional allele (PAV/PAV) sense PTC/6-PTU to be intensely bitter (known as super-taster phenotype), while individuals possessing two non-functional alleles (AVI/AVI) are the least bitter-sensitive (non-taster phenotype). In contrast, heterozygotes (PAV/AVI) show a wide range of bitter taste perception (often described as medium-tasters) [41]. Thus, tasters show lower consumption of several bitter vegetables, such as those from *Brassicaceae* family (rich in a group of thiourea-containing compounds) [42, 43]. In other investigations, two polymorphisms rs10772423 (Val240Ile) and rs10845293 (Ala227Val) located in TAS2R31 gene (formerly TAS2R44) were associated with the bitter compounds amarogentin and grosheimin intensity ratings, detection and recognition threshold, Q bitterness and grapefruit liking [16, 44]. Moreover, Val240 homozygotes tended to be less sensitive to bitterness from artificial sweetener acesulfame potassium than the Ile240 homozygotes [45]. Analogously, TAS2R19 SNPs rs10772420 and rs1868769 were also found to influence Q and grosheimin intensities along with detection and recognition threshold by five studies [16, 44, 46–48]. Much less is known about the effect of the genetic alterations of other TAS2Rs, which proteins also function as bitter taste receptors. It cannot be ruled out that not all TAS2R gene polymorphisms are as consequential as the abovementioned.

### The singaling pathways downstream of TAS2Rs in response to stimulatory signals

**Fig. 2:**
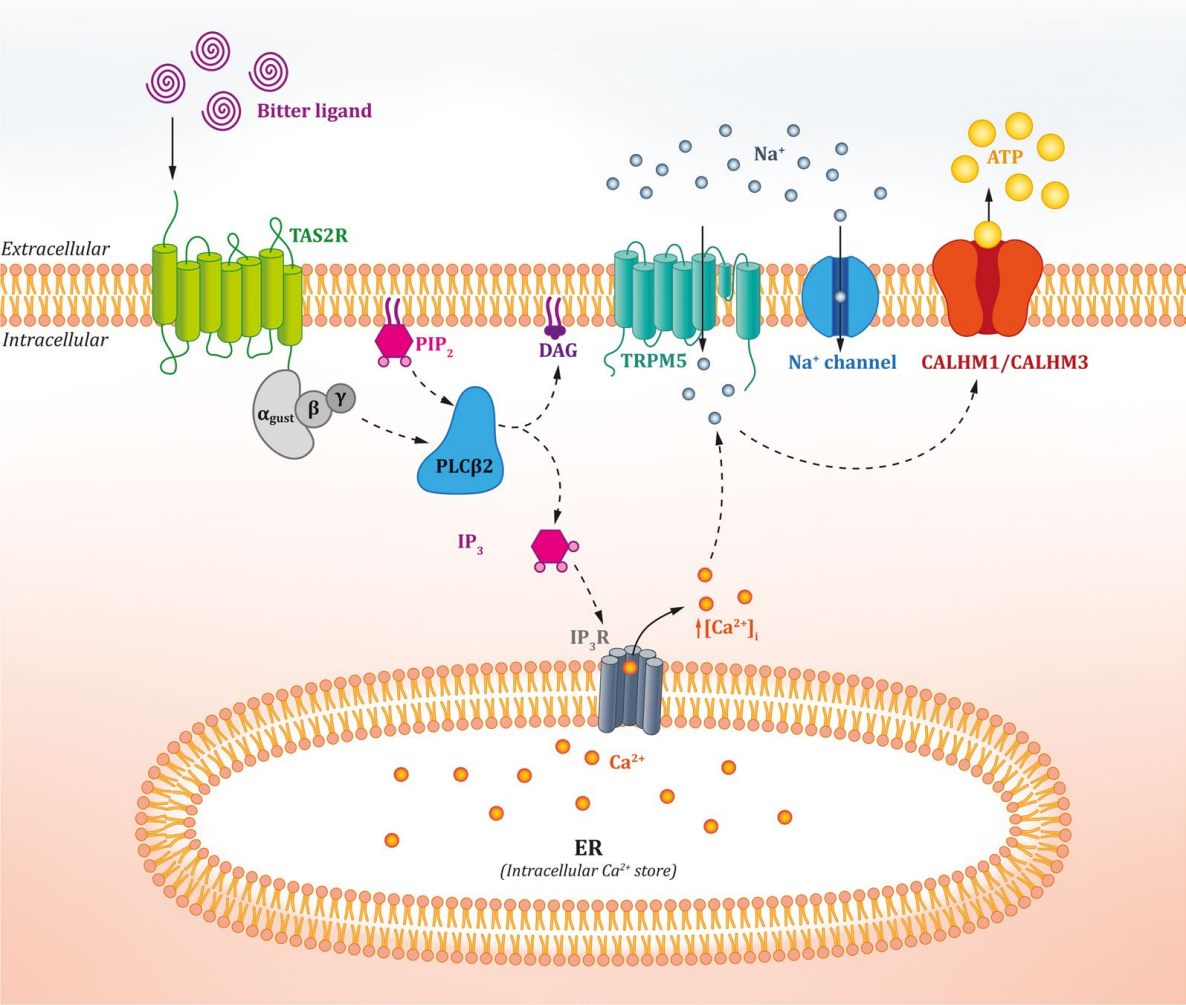
Overview of the canonical TAS2R signalling pathway. Upon TAS2R activation, the G-protein gustducin dissociates and activates phopholipase Cβ2 (PLCβ2), which subsequently leads to elevation in Ca^2+^ from inositol-1,4,5-triphosphate (IP3)-sensitive Ca^2+^ stores. This in turn activates transient receptor potential cation channel subfamily M member 5 (TRPM5), alters membrane potential (depolarisation), and triggers the release of the neurotransmitter, adenosine-5'-triphosphate (ATP), via the calcium homeostasis modulator 1 CALHM1 or CALHM1/CALHM3 ion channel. All these events together contribute to a wide range of cellular response

As shown in Fig. 2, the canonical taste signal transduction pathway, common for bitter, sweet, and umami taste receptors [49], is based on the dissociation of the activated heterotrimeric G-protein α-gustducin [50] and the further activation of phopholipase Cβ2 (PLCβ2) [49, 51]. Studies have shown that mice lacking genes encoding α-gustducin [52, 53] or PLCβ2 [49, 54] are characterized by dispersion of bitter, sweet, and umami tastes, with undisturbed perception of salty and sour tastes, which proves that different signalling pathways are responsible for the above taste. PLCβ2 hydrolyses phosphatidyliinositol-4,5-bisphosphate to diacylglycerol and inositol-1,4,5-triphosphate (IP_3_), which binds to the inositol triphosphate receptor (IP_3_R_3_). After binding, IP_3_ generates a calcium wave ([Ca^2+^]_i_), which involves the influx of Na^+^ ions through activated transient receptor potential cation channel subfamily M member 5 (TRPM5) [49, 55]. Some investigators believe that in addition to TRPM5-dependent mechanisms, TRPM5-independent mechanisms are responsible for the transduction of taste signals [56, 57], which involve, for example, TRPM4 [58]. Cells respond to sodium signal via depolarisation and extracellular efflux of the neurotransmitter, adenosine-5'-triphosphate (ATP), through the calcium homeostasis modulator 1 (CALHM1) ion channel [59]. Recent studies have highlighted another possible mechanism for the release of ATP after sodium activation, which involves the participation of the CALHM1/CALHM3 ion channel [60]. The released neurotransmitter re-activates ATP-gated ionotropic receptors, P2RX2 and P2RX3, on afferent flavour fibres [61, 62], as well as P2Y metabotropic receptors found in type II and III TRCs [63–65]. However, it is noteworthy that non-lingual TAS2Rs may trigger tissue-specific signalling pathways to execute different biological roles depending on cell types. In addition to initiation of bitter taste perception, few additional transduction cascades following Ca^2+^ signalling lead to muscle relaxation [66–73] and contraction [72, 73].

## Extra-oral expression of the bitter taste—role in (patho)physiological processes

### The respiratory system

The respiratory tract organs connect the human body to the external environment. Hence, it is constantly exposed to toxic dusts or aerosols. Therefore, the fundamental role of the respiratory tract is the early prevention of adhesion, surface colonisation, and biofilm formation by microorganisms, and the effective removal of harmful substances via diverse defence mechanisms that are subject to complex regulatory processes. In addition to nerve and hormonal interactions, local regulatory mechanisms also integrate the respiratory tract functions with the host defence pathway [74]. Hence, TAS2Rs identified in multiple airway cell types, including airway smooth muscle cells (ASM), distinct epithelial cell subtypes, as well as resident (macrophages) and migratory hematopoietic (neutrophils, mast cells, and lymphocytes) inflammatory cells, may be particularly important.

#### Solitary chemosensory cells

The distribution of various TAS2R subtypes and their downstream signalling components (such as α-gustducine, PLCβ2, TRPM5, and IP_3_R_3_) in solitary chemosensory cells (SCCs) of the paranasal sinus epithelium has been confirmed in rodents [11] and humans (Tables 1, 2) [75–77]. Activation of these chemoreceptors on SCCs by different bitter ligands (including bacterial AHL-12) triggers robust α-gustducine-, PLCβ2- and TRPM5-dependent stimulation of acetylcholine-sensitive peptidergic trigeminal nerve fibres (via the nicotinic acetylcholine receptor, NACh -R) to secrete the calcitonin gene-dependent peptide and substance P. Substance P, in turn, activates neurokinin 1 receptors (NK_1_R) in endothelial and mast cells, leading to their degranulation and plasma extravasation, which elicited the development of so-called neurogenic inflammation to prevent bacterial infections [75]. Furthermore, in rodents, sensitisation of these trigeminal nerve fibres by bitter tastants induced reflexive respiratory rate depression (up to apnoea), thereby limiting continued inhalation of toxic dusts and microbes [11, 78]. In the human sinus SCCs, hTAS2R47 agonists (such as denatonium benzoate [DB]) stimulate a [Ca^2+^] wave within the cell, which propagates into adjoining cells via gap junctions and elicits the release of antimicrobial peptides (AMPs), including β-defensins [77]. Furthermore, AMP secretion after TAS2R stimulation occurred rapidly (~ 5 min) compared to enhancement in AMP production over hours in response to Toll-like receptor (TLR) stimulation [77]. In addition to TAS2Rs, SCCs have also been reported to co-express sweet taste receptors (T1R2/T1R3). More importantly, sweet and bitter signals oppositely regulate innate immunity. Sugars (such as glucose and sucrose) present in the upper airway surface liquid, inhibit bitter taste-induced calcium release. Consequently, downstream, calcium-driven initiation of the innate immune system at the tissue level (such as release of antimicrobial components) is impaired [77]. This may potentially explain the enhanced susceptibility to bacterial respiratory infections in patients with chronic rhinosinusitis (CRS), in whom high levels of glucose have been detected in nasal secretions due to the dysfunction of the mucosal epithelium and initiation of the inflammatory cascade [79]. Owing to a similar mechanism [80], patients with prediabetes or diabetes can be more severely affected by different respiratory infections [81]. Recent studies have also suggested that glucose levels in the airways rapidly are depleted during a bacterial infection due to the bacterial load [82].

**Table 1:** Expression and distribution of TAS2Rs in different normal human tissue types

System		Receptor type																													Methods	Refs.
		1	3	4	5	7	8	9	10	13	14	16	19	20	30	31	38	39	40	41	42	43	44	45	46	47	48	49	50	60		
Respiratory system	SCCs			+							+														+	+					IFC,RT-PCR	[76, 77]
	HSECs			+								+					+														IFC, IHC	[27, 76]
	ASM	+	+	+	+	+	+	+	+	+	+		+	+	+	+	+	+			+	+		+	+				+		IFC, RT-PCR	[60, 62]
Immune system	CD34 + stem cells																+														FC	[116]
	Mast cells		+	+	+				+	+	+		+	+											+						RT-PCR	[111]
	Monocytes	+	+	+	+	+	+	+	+	+		+	+	+	+	+	+	+	+	+	+	+		+	+				+	+	ICC, RT-PCR, qRT-PCR, FC	[109, 111, 112]
	Macrophages		+	+	+	+	+	+	+		+		+	+		+	+	+				+		+	+						IHC, IFC, RT-PCR, FC	[111, 113, 120]
	Neutrophils	+	+	+	+	+	+	+	+	+	+	+	+	+	+	+	+	+	+	+	+	+		+	+				+	+	ICC, IFC, RT-PCR, qRT-PCR, FC	[113–116]
	B cells	+	+	+	+	+	+	+	+	+	+	+	+	+	+	+	+	+	+	+	+	+		+	+				+	+	ICC, RT-PCR, qRT-PCR	[113]
	T cells	+	+	+	+	+	+	+	+	+	+	+	+	+	+	+	+	+	+	+	+	+		+	+				+	+	ICC, RT-PCR, qRT-PCR	[113]
	NK cells	+	+	+	+	+	+	+	+	+	+	+	+	+	+	+	+					+		+	+				+	+	ICC, RT-PCR, qRT-PCR	[113]
	Bone marrow (MSCs)																								+						MS, IHC, IFC, RT-PCR, FC	[70]
Cardio-vascular system	Heart	+	+	+	+	+	+	+	+	+	+	+	+	+	+	+	+	+	+	+	+	+		+	+				+	+	ISH, qRT-PCR	[202, 203]
	Arteries	+	+	+	+	+	+	+	+	+	+							+	+		+								+	+	FC,IFC, RT-PCR, qPCR, WB	[64–66, 207]
Endocrine system	Thyroid	+	+	+	+	+	+	+	+	+	+	+		+	+	+	+	+	+	+	+	+		+	+		+		+	+	IHC, RTPCR	[215]
Reproductive system	Placenta										+						+														IHC, IFC, RT-PCR	[124, 125]
	Myometrium		+	+	+	+	+		+	+	+					+		+			+	+		+					** + **		IHC, qPCR	[134]
	Testis and sperms		+	+							+		+									+									ddPCR, IFC, WB	[137]
Digestive system	EECs		+	+	+												+														IHC, PCR, RT-PCR, qRT-PCR	[169, 171, 176]
	Gastrointestinal smooth muscle cells		+	+					+																						RT-PCR	[175]
Excretory system	DMS	+		+	+	+	+	+	+	+	+			+	+	+	+	+	+					+					+		qRT-PCR	[73]
Nervous system		+	+	+	+	+	+	+	+	+	+	+		+			+	+	+	+	+	+	+	+	+		+		+	+	Microarray data analysis, ICC, IFC, IHC, RT-PCR, WB	[193]
Adipose tissue																	+														qRT-PCR, WB	[219]
Skin		+	+	+	+	+	+	+	+	+	+	+	+	+	+	+	+	+	+	+	+	+		+	+				+	+	IHC, RT-PCR, WB	[125, 221, 222]

*ASM airway smooth muscle, *ddPCR* droplets digital polymerase chain reaction, *DMS* detrusor smooth muscle, *EECs* enteroendocrine cells, *FC* flow cytometry, *HSECs* human sinonasal epithelial cells, *ICC* immunocytochemistry, *IFC* immunofluorescence, *IHC* immunohistochemistry, *ISH* in situ hybridisation, MS mass-spectrometry, *MSCs* mesenchymal stem/stromal cells, *NK* natural killer *RT-PCR* reverse transcription-polymerase chain reaction, *qRT-PCR* quantitative reverse transcription-polymerase chain reaction, *SCCs* solitary chemosensory cells, *WB* Western blotting

**Table 2:** Expression and distribution of TAS2Rs in different normal mouse tissue types

System		Receptor type																																	Methods	Refs.
		102	103	104	105	106	107	108	109	110	113	114	116	117	118	119	120	121	122	123	124	126	129	130	131	134	135	136	137	138	139	140	143	144		
Respiratory system	SCCs										+																			+					ICC, ISH	[11]
Immune system	Thymus				+			+																	+										IHC, ISH, qRT-PCR	[120, 122]
Cardio-vascular system	Heart														+							+					+						+		ISH, qRT-PCR	[202, 203]
	Arteries												+																				+		FC, IFC, RT-PCR	[70]
Reproductive system	Testis and sperms	All 35 mTAS2Rs																																	ICC, IHC, ISH, RT-PCR, qRT-PCR	[134, 135]
Digestive system	Tuft cells														+																				RNAscope hybridisation and imaging	[139]
	Paneth cells																								+										IHC, ISH, RT-PCR	[147]
	Goblet cells																								+										IHC, ISH, RT-PCR	[147]
	EECs	+		+	+	+	+	+		+	+	+	+	+		+		+	+	+	+	+	+	+		+	+	+	+	+	+	+	+	+	ICC, RT-PCR	[159, 164]
	Gastrointestinal smooth muscle cells							+																					+						RT-PCR	[175]
Excretory system	Kidney	+								+	+		+		+							+	+	+	+		+	+	+	+	+	+	+	+	IHC, IFC, RT-PCR, qRT-PCR, WB	[209–211]
	DMS	+	+					+	+		+	+	+	+	+	+	+		+			+		+	+		+		+			+	+	+	qRT-PCR	[73]
Nervous system													+																						IHC, ISH, RT-PCR	[116, 191]
Adipose tissue										+	+				+	+						+				+	+		+	+		+	+	+	RT-PCR	[216]

*DMS detrusor smooth muscle, *EECs* enteroendocrine cells, *FC* flow cytometry, *ICC* immunocytochemistry, *IFC* immunofluorescence, *IHC* immunohistochemistry, *ISH* in situ hybridisation, *RT-PCR* reverse transcription-polymerase chain reaction, *qRT-PCR* quantitative reverse transcription-polymerase chain reaction, *SCCs* solitary chemosensory cells, *WB* Western blotting

#### Human sinonasal epithelial cells

Within the nasal cavity, human sinonasal epithelial cells (HSECs) have also been shown to express multiple TAS2R isoforms, which form a repertoire different from that of denatonium-responsive SCCs, indicating functional diversity between these two types of cells (Table 1) [30, 83]. Over 96% cilia of HSECs express hTAS2R38, which is not found on SCCs [30]. In addition, they express hTAS2R4 and 16, encoded by genes on chromosome 7 near the *hTAS2R38* site, which are paradoxically stimulated also by DB [33]. Therefore, epithelial TAS2R-mediated detection of the molecules secreted by pathogens appears to play an important role in airway immunity. The proximal signalling events that follow hTAS2R38 activation by AHLs [30] or quinolones [32] derived from Gram-negative bacteria, such as *Pseudomonas aeruginosa,* in HSECs are marked by nitric oxide (NO) production in [Ca^2+^]-, PLCβ2-, TRPM5-, and IP_3_R-dependent manner. NO, in turn, increases ciliary beat frequency (CBF) and subsequently mucociliary clearance of the inhaled pathogens, indicating bactericidal effect of TAS2Rs [30]. Furthermore, quorum detection molecules (different from AHLs) released by Gram-positive bacteria, such as methicillin-resistant *Staphylococcus aureus* [33], *Staphylococcus epidermidis* [34], or *Bacillus cereus* [35], also demonstrated NO-dependent defence response, similar to what has been shown for Gram-negative bacteria, which is completely or partially independent of the TAS2Rs. This may be because polymorphisms in *TAS2R38* have been linked to NO synthesis rate, its bactericidal activity, and susceptibility to upper respiratory tract infections, including CRS. Preliminary in vitro studies have shown that homozygosity for the *TAS2R38* functional genotype (PAV) is required for maximal T2R38 signal propagation in response to AHLs in sinonasal respiratory epithelial cells [30]. This correlation was confirmed in further clinical trials conducted mainly by Cohene's group and others [84–88]. Over 90% patients with CRS undergoing functional endoscopic sinus surgery were carriers of the non-functional *TAS2R38* genotype (n = 28: 3.6% PAV/PAV, 50% PAV/AVI, 46% AVI/AVI *vs.* population *TAS2R38* genotype distribution in Europeans: 20% PAV/PAV, 50% PAV/AVI; 30% AVI/AVI; *P* < 0.043 in the χ^2^ test] [84, 89]. The PAV/PAV homozygotes experienced threefold improvement after surgery compared to those who harboured at least one AVI allele [87]. It is noteworthy that this association was not observed in Italian patients [90]. This discrepancy may partially stem from differences in the experimental model used, as the above study recruited patients who were clinically more resistant to treatment and showed stronger T helper type 2 (Th2) immune response than those in previous studies. Among known CRS risk factors, biofilm formation significantly contributes to chronic mucosal inflammation [91]. Thus, some have shown that bacterial biofilms were more frequent in sinonasal specimens from TAS2R38 non-tasters than in tasters or supertasters (*P* < 0.019) [92], which confirms the negative association previously demonstrated in vitro by Adappa et al*.* [93]. CRS is also nearly ubiquitous in patients with cystic fibrosis (CF) [94]. Interestingly, a recent study has demonstrated that the frequency of the PAV allele of *TAS2R38* was significantly reduced not only in the CF population with nasal polyps requiring surgery, but also in CF patients with chronic pulmonary colonisation by *P. aeruginosa.* These data suggest a role for TAS2R38 as a novel modifier of sinonasal disease severity and of pulmonary *P. aeruginosa* colonisation in people with CF [95]. Similar to that observed in the upper respiratory tract, TAS2R-dependent defence mechanisms were detected in its lower parts [96, 97].

#### ASM

The presence of TAS2Rs in ASM (Tables 1, 3), confirmed for the first time in 2010 by Desphande et al., has set a new trend in scientific research, especially in the field of pulmonology. Three of the 25 TAS2Rs (TAS2R10, 14, and 31) were expressed four times more than β_2_-adrenoreceptors (β_2_ARs) [66]. The ASM-relaxing effect of activated TAS2Rs has been unequivocally established in distinct species (mice, guinea pigs, and human) using isolated airways and lung slices [66, 68, 98]. Bitterants, such as ChQ, elicited TAS2R-mediated relaxation with similar efficacy as the β_2_ agonist, isoproterenol, in human ASMs [99]. These render TAS2R agonists attractive bronchodilators. Importantly, the ASM-relaxing TAS2Rs displayed strong adjuvant activity in mice, particularly abetting β-agonist-induced relaxation, suggesting that bitter tastants can be used for adjunct therapy to standard bronchial asthma treatment [66]. This is additionally supported by the fact that TAS2R expression, signalling, or bronchodilating function were not down-regulated by airway inflammation in ASM cells from asthmatic patients and human lung preparations [100]. Most importantly, although these chemoreceptors, as representatives of the GPCR family, are hypothetically predisposed to tachyphylaxis, repetitive exposure to Q only slightly reduced its effectiveness in terms of [Ca2^+^]_i_ release and ASM relaxation, probably due to G-protein-coupled receptor kinases (GRKs) [100]. Under conditions of marked desensitisation of β_2_AR-mediated relaxation response induced by prolonged β-agonist treatment, TAS2R agonists showed complete efficacy [101]. This further suggests the superiority of a pure dual bronchodilator approach based on TAS2R/β_2_AR agonists acting via different mechanisms over monotherapy. However, as in the case of β_2_ARs [102], at least one study postulates genetic variations in TAS2Rs as valuable markers for predicting therapeutic response and outcomes in patients with asthma [103].

**Table 3:** Expression and distribution of TAS2Rs in different normal tissue types of other species

System		Receptor type	Methods	Refs.
Respiratory system	ASM	pTAS2R3,-4,-10	RT-PCR	[98]
Cardio-vascular system	Heart	rTAS2R13,-38,-40,-103–110,-113,-114,-116–121,-123–126,-129,-130,-134–137,-140,-143	ISH, qRT-PCR	[203]
	Arteries	rTAS2R39,-40,-108,-114,-130,-137,-140	IFC, RT-PCR, WB	[208]
Nervous system		rTAS2R4,-10,-38,-109,-144	IHC, IFC, RT-PCR, WB	[189– 191]

*ASM airway smooth muscle, *IFC* immunofluorescence, *IHC* immunohistochemistry, *ISH* in situ hybridisation, *pTAS2Rs* pig bitter taste receptors, *rTAS2Rs* rat bitter taste receptors, *RT-PCR* reverse transcription-polymerase chain reaction, *qRT-PCR* quantitative reverse transcription-polymerase chain reaction, *WB* Western blotting

While the bronchodilatory effect of bitter substances is a generally accepted consensus, the underlying mechanism is controversial. Initially, Desphande et al. suggested that paradoxically, it is a consequence of Gβγ-, PLCβ2- and IP_3_-dependent local growth of [Ca^2+^]_i_ to a level at which bronchoconstriction caused by typical airway constrictors, such as acetylcholine (Ach) or histamine, is expected. The elevation in [Ca^2+^]_i_ subsequently leads to activation of large conductance KCa^2+^ channels (BK_Ca_) (but also inhibits Ca^2+^ sensitisation and actin polymerisation), membrane hyperpolarisation, and bronchodilation [66]. Conflicting reports, however, revealed that bronchodilation is rather a response either to inhibition of intracellular calcium oscillations, reduced Ca ^2+^ sensitivity in ASM [104], or blockage of the voltage-dependent L type alpha 1C subunit of the calcium channel (Cav1.2) [67]. Grassin-Delyle et al. hypothesised the contribution of the phosphoinositide 3-kinase (PI3K) signalling pathway [68], while according to Tazzeo’s group, bitter substances (such as caffeine) induce ASM relaxation by triggering direct depolymerisation of actin [69]. It is noteworthy that the in vitro relaxation of guinea pig trachea in response to different bitterants was highly dependent on the precontraction agents used, suggesting differential regulation of compartmentalised signalling in ASM cells [98].

A specific morphological feature of bronchial asthma is the reconstruction of the airways, which in some patients may lead to irreversible bronchoobturation. Although controversial, airway remodelling is commonly attributed to the underlying increased proliferative activity of ASM cells under the influence of GPCR agonists, cytokines, chemokines, and growth factors released in the bronchial tree due to allergen-induced airway inflammation [105]. While contemporary pharmacotherapy of bronchial asthma alleviates chronic inflammation, remodelling remains a serious problem in therapeutic management of this disease [106]. Recently, selected bitter substances have been shown to exert an antimitogenic effect on primary human ASM (healthy and asthmatic) cells in a concentration-dependent manner [107, 108], and the participation of TAS2Rs in the above mechanism was confirmed via small interfering RNA (siRNA)-mediated silencing of their genes [108]. Simultaneously, cell cycle progression in human ASM cells was found to be inhibited by ChQ and Q, presumably due to PI3K inhibition, which is involved in the induction of the pro-mitotic signal [107]. In addition, the above-mentioned compounds impaired the structure and function of mitochondria, leading to mitophagy and subsequent ASM cell death via the proapoptotic BCL2interacting protein 3 (BNIP3) [109]. The promising results of the above study suggest that TAS2Rs can be an interesting therapeutic option in future, the goal of which would be to repair, regenerate, and/or replace damaged airway tissue structures.

### The immune system

Perhaps the most surprising revelation regarding TAS2Rs was their expression in immune cells, from the hematopoietic CD34^+^ stem cells to tissue resident macrophages and infiltrants, such as monocytes and neutrophils (Table 1) [110–116]. Thus, it is becoming increasingly clear that their roles in airway epithelial innate immunity may only represent the “tip of the iceberg” regarding the importance of bitter chemoreceptors in immunity.

#### The innate immunity

Supporting the above observations, the distribution of TAS2Rs, including those of the 31, 38, and 43 subtypes, was recently demonstrated in polymorphonuclear neutrophils that represent the earliest bona fide innate immune cells recruited to the site of inflammation. They respond to AHL-12 stimulation with transmigration, overexpression of adhesion receptors (CD11b), chemotaxis, and phagocytosis [113–115]. Further testing clarified that mTAS2R138 facilitate the degradation of lipid droplets (LDs) in neutrophils during *Pseudomonas aeruginosa* infection through competitive binding with peroxisome-proliferator-activated receptor γ (PRAR γ) antagonist, aforementioned AHL-12. The released PRAR γ then migrates from nuclei to the cytoplasm in order to accelerate the degradation of LDs by binding to perilipin-2. Subsequently, the mTAS2R138-AHL-12 complex targets LDs to enhance their degradation, and thereby facilitating the clearance of AHL-12 in neutrophils to maintain homeostasis in the local environment [29].

Other important players in early innate immune responses have been also reported to express TAS2Rs, namely primary human monocyte-derived unprimed (M0) macrophages (MΦs). TAS2R agonists “rev up” acute phagocytic activity via calcium-driven NO-activation of guanylyl cyclase to increase cyclic guanosine monophosphate level [117]. Interestingly, as mentioned earlier, similar intracellular signalling pathway was detected in airway epithelial cells [30, 33–35], although the physiological output was different. In ciliated cells, the mechanism involved controlling the CBF, while it regulated phagocytosis in MΦs. Both processes are critical for innate defence, emphasising a role of TA2Rs as immune recognition receptors for chemosensing bacteria. Mesenchymal bone marrow cells (MSCs) have been reported to exhibit immunomodulatory effects on both humoral and cellular components of the innate immune system [118]. In these cells, the presence of hTAS2R46 was demonstrated using a large mass spectrometry-based proteomic screening. Stimulation of these receptors activates intracellular calcium signals that reduce the level of 3',5'-cyclic adenosine monophosphate to increase extracellular secretion of ATP [70]. Thus, TAS2Rs may be assumed to affect the immunomodulatory potential of MSC, which should be verified in future.

#### The adaptive immunity

Emerging evidence also supports the hypothesis that in addition to innate immunity, TAS2Rs function as a novel arm of the adaptive immune response. Some human resting or activated lymphocytes have been found to express multiple TAS2R isoforms and respond to multiple types of bitter agonists. Importantly, lymphocytic hTAS2R38 expression, analogous to that in monocytes and neutrophils [116], was age-dependent, with significantly higher levels in the younger age group (20–35 years) than in the older group (60–90 years) [114]. Considering these results from the viewpoint of immune system remodelling in the elderly, it is perhaps not surprising that TAS2R38 down-regulation may be a mechanism underlying its impairment [119]; however, this requires further investigations. Each of the peripheral blood lymphocyte subpopulations were characterized by heterologous expression profile of hTAS2R38. Unlike that in CD19^+^ B lymphocytes, TAS2R38 was expressed significantly in CD3^+^ lymphocytes (fold change in expression level: 9.66 vs. 17.28), among which CD4^+^ T helper cells (Th) showed higher level of expression than CD8^+^ cytotoxic T cells (Tc) (fold change in expression level: 24.12 vs. 16.07). Further subpopulations of T lymphocytes expressing hTAS2R38 include naive (Tn), as well as central (Tcm) and effector (Tem) memory cells [116]. Interestingly, Tran et al. observed higher expression of hTAS2R38 in T lymphocytes expressing early (CD69^+^) and late (CD25^+^) activation markers than in the cells that stained negative for those antigens suggesting that their stimulation and differentiation states may act as “rheostat” to control the magnitude of the TAS2R response [116]. Lymphoid progenitors that have developed from hematopoietic stem cells in the bone marrow migrate to the thymus to complete their antigen-independent maturation into functional T cells. Studies on a mouse experimental model have identified mTAS2R131, along with its downstream signalling elements (α-gustducine, PLCβ2, and TRPM5) in the thymus core [120], mainly in thymic cholinergic chemosensory cells (CCCs) (Table 2) [121, 122]. The role of these receptors in the thymus remains unclear, although it may contribute to the aforementioned T cell development.

#### Anti-inflammatory activity

In addition to being the “engines” stimulating host defences, TAS2Rs have also long been known to exert immunosuppressive activities, such as reduction in the synthesis of inflammatory cytokines by various cell types, including human macrophages or monocytes. This is enhanced by exposure of the cells to bitter saikosaponin B extracted from *Bupleuri radix* (1) or goitrin (2), as is evident from the inhibition of IgE-dependent mast cell degranulation (1) or reduction in secretion of pro-inflammatory TNF-α from peripheral blood mononuclear cells via hTAS2R38 (2), respectively [116, 123]. This inflammatory licensing possibly correlates with genetic polymorphisms of TAS2Rs, as it was not observed in the non-functional diplotype, AVI/AVI [116]. Genome-wide expression array analyses on peripheral blood leukocytes obtained from children with severe therapy-resistant asthma also support the anti-inflammatory theory, reporting profound up-regulation of TAS2Rs associated with reduced secretion of several pro-inflammatory mediators under the influence of bitterants, ChQ, and DB [110]. Similarly, in human lung macrophages isolated from patients undergoing surgery for carcinoma, different TAS2R subtypes were possibly involved in the inhibition of lipopolysaccharide (LPS)-induced cytokine production, with maximal level of inhibition (≥ 90%) observed with Q, ChQ, phenanthroline, and erythromycin, similar to that observed for 10^–8^ M budesonide [124].

### The reproductive system

#### Placenta

**Fig. 3:**
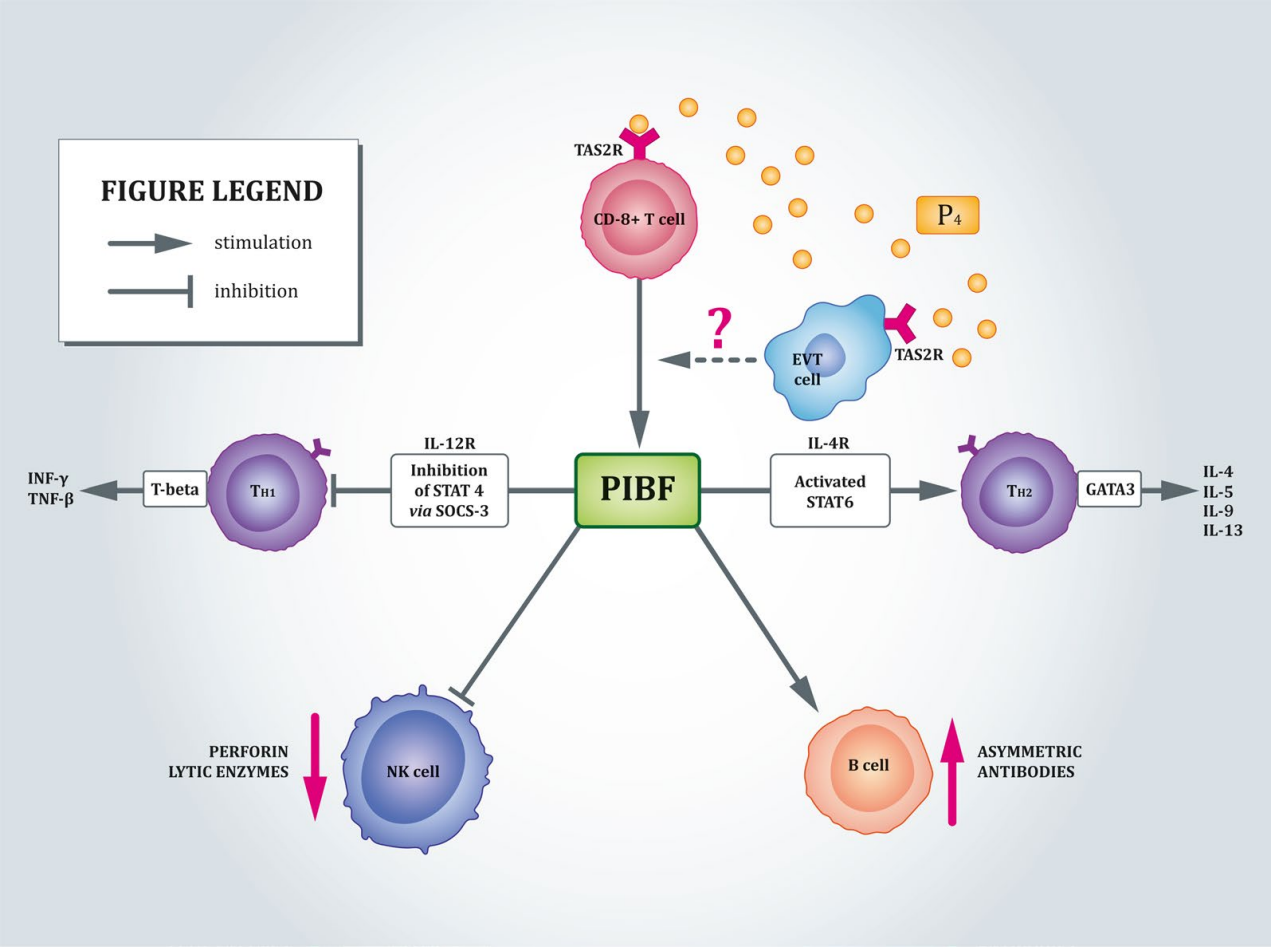
Model showing TAS2R-dependent modulation of progesterone (P4)’s role in protecting from pregnancy. P4 binds to TAS2Rs found in extravillous trophoblast (EVT) cells and enhances the release of progesterone-induced blocking factor (PIBF), perhaps via the canonical signalling cascade. This, subsequently binds to the PIBF receptor, which heterodimerises with the IL-4 receptor to activate the signal transducer and activator of transcription 6 (STAT6) transcription pathway for cytokine production by the type 2 T helper cells (Th2). On the type 1 T helper cells (Th1), PIBF acts to inhibit the STAT4 pathway by promoting interaction of suppressor of cytokine signalling 3 (SOCS-3) with the IL-12R receptor to attenuate Th1 cytokine production. Furthermore, PIBF inhibits the cytological activity (natural killer) of NK cells by blocking perforin and lytic enzyme degranulation. Finally, PIBF interacts with B cells, increasing the production of asymmetric antibodies. All this reduces cellular cytotoxicity and controls immunosuppression

In addition to their localization in the oral cavity and respiratory tract, it is now evident that TAS2Rs are also present in tissues and organs, which initially were not known to harbour these receptors, such as the reproductive system. Strong TAS2R14 and 38 expression was detected in human placental tissues, as well as in several choriocarcinoma trophoblast‐derived cell lines and HTR‐8/SVneo, an immortalized first trimester placental trophoblast cell line (Table 1) [125, 126]. In addition, TAS2R14 was co-expressed with cholecystokinin (CCK) [126]. Both TAS2R-specific agonists notably increased intracellular calcium level in these human trophoblast cell lines [125, 126]. The physiological role(s) of these receptors in normal and pathological placentas remain largely unknown; however, based on analogy with other organ systems, we speculated that they may contribute to immunological regulation at the maternal‐foetal interface [126]. In fact, throughout human pregnancy, dynamic local and systemic immune changes confer tolerance to the genetically ‘foreign’ semi-allogeneic foetus, while protecting the mother against invading pathogens [127]. The appropriate execution of these important events requires a keystone “pacemaker” for pregnancy, namely, progesterone (P4). P4 influences the pregnancy timeline via its immunomodulatory activity, which is mediated via dampening of type 1 or pro-inflammatory reactivity and augmentation of type 2 or anti-inflammatory immunity [128, 129]. Considering that P4 is both mTAS2R110 and 114 agonist [38], it is tempting to hypothesise that TAS2R signalling in the trophoblast involves mechanisms via which this steroid hormone modulates the immune system during pregnancy, as shown in Fig. 3. Thus, further studies are required to adequately decipher the complex role of the receptors in the immune clock during pregnancy. Other possible functions are related to TAS2R-mediated pathogen recognition. As mentioned above, TAS2R38 detects physiological concentrations of AHLs to activate calcium-dependent NO-production, indicating its role as a sentinel receptor to identify bacteria and regulate innate immune responses in airway ciliated cells [114] and MΦs [117]. Conceivably, a similar mechanism operates in the syncytiotrophoblast (ST) or amnion (both of which protect the embryo), ultimately acting as a sensor for bacterial infection and contributing to inter-kingdom communication within the placenta [125, 126]. Possibly, at least some placental TAS2Rs may also detect bitter components or products secreted by pathogenic viruses or fungi, which requires further investigations. Furthermore, amniotic cells respond to microbial challenge by releasing natural AMPs [130]; therefore, the TAS2R-dependant machinery may aid in this bactericidal activity, analogous to the SCCs in the airways [77]. Apart from AMPs, the placenta is also an important source of ATP-binding cassette (ABC) transporters, including P-glycoprotein (P-gp; encoded by *ABCB1*) and breast cancer-related protein (BCRP; encoded by *ABCG2*). They utilize ATP hydrolysis to translocate a wide variety of endo- and exogenous substrates, thereby preventing cell compartments from accumulating detrimental toxins and metabolites [131, 132]. In TAS2R38-expressing enteroendocrine cells (EECs), “potentially toxic” bitter substances that are not completely excreted stimulate CCK secretion. This, in turn, enhances ABCB1 expression, while increasing toxic compound extrusion into the intestinal lumen [133]. Hence, TAS2Rs anchored in syncytiotrophoblast (ST) and extravillous trophoblast (EVT) possibly provide a sentinel line of defence that protects the placenta from potentially harmful agents, thereby safeguarding the developing foetus [126]. Last but not least, bitter GPCRs may be one of the most promising chemodetectors controlling the synthesis and secretion of other placental hormones or active factors critical for human foetal growth and development.

#### Myometrium

Interestingly, uterine smooth muscle cells from mouse and humans also express TAS2Rs and their canonical signalling components (such as α-gustducin and PLCβ2) (Table 1). Bitter tastants (ChQ, DB, and 1,10-phenanthroline) completely relaxed uterine strips pre-contracted using different uterotonics (oxytocin, KCl, and prostoglangide F2 α [PGF2α]) in a concentration-dependent manner, and this effect was higher than that observed after using common tocolytics (nifedipine, indomethacin, and MgSO_4_). Knock-down of hTAS2R14, but not that of hTAS2R10, in human myometrium cells inhibited ChQ’s ability to reverse an oxytocin-induced rise in [Ca^2+^]_i_ and myometrial contraction [134]. Another attractive characteristic of bitterants (such as ChQ) is that they can prevent mouse preterm birth induced by bacterial endotoxin LPS or nuclear progesterone receptor antagonist RU486 more often than clinically used tocolytics, and α-gustducin can mediate ChQ-induced protection [134]. Hence, as bitter tastants and current tocolytics act on different molecular targets, TAS2Rs can act as druggable targets to promote uterine quiescence and reduce the number of preterm births. However, more studies are required to better elucidate the mechanism underlying the tocolytic action of TAS2Rs in mouse and human uteri.

#### Testis and sperm

A large number of genes encoding the identified TAS2Rs, along with the entire bitter taste transduction cascade, are expressed in mouse [135, 136] or human [137] testis and sperm (Tables 1, 2), indicating the involvement of these receptors in male gametogenesis and fertility. This is supported by the reproductive phenotype of mouse harbouring a targeted deletion of taste receptor genes [136], in addition to a significant correlation between human semen parameters and their polymorphic variants [138]. However, TAS2Rs in sperm and testicular tissue may also play more defensive and protective roles, as clearly established for gustatory cells, where their activation instantly actuates an innate aversion against the ingestion of potentially toxic bitter compounds. Indeed, sperm cells display heterogeneous expression profile of individual TAS2R subtypes, presumably to distinguish among different bitter stimuli, thereby representing specialized molecular sensors fundamental for sperm chemosensation and/or guidance [135]. Therefore, in-depth research is required to determine the actual contribution of TAS2Rs to sperm physiology.

### The digestive system

The gastrointestinal tract (GI), the core interface between ingested meal and the human body, plays a critical role in “tasting” luminal stimuli and eliciting adequate motor and secretory responses, as well as assimilating macronutrients and eliminating waste products. Harmful contaminants in food also initiate protective response, such as inhibition of gastric emptying, vomiting, diarrhoea, or food aversion, to reduce, reject, or avoid further intoxication. The chemosensory networks involved are similar to those present in the lingual system, and are found on several cell types in the gut epithelium, such as enterocytes, EECs, tuft cells, Paneth cells, goblet cells, microfold cells, and cup cells.

#### Tuft, Paneth and goblet cells

TAS2Rs and the downstream taste signalling molecules (α-gustducine, α-transducine, and TRPM5) in intestinal tuft cells (Table 2) [139–142] are being actively investigated, prompted by the recent observations regarding unsuspected connections to protective type 2 immunity. These receptors constitute a conduit via which ligands from the parasitic helminths are sensed to generate outputs unique among epithelial cells, the cytokine IL-25 being crucial in driving the characteristic “allergic” immune response. A tuft cell-initiated IL-25-driven positive feed-forward loop resulted in type 2 innate lymphoid cell (ILC2) expansion, along with IL-13-driven tuft and goblet cell hyperplasia, presumably via stem cell niche signalling [143–145]. Goblet cells provide the mucous component of the protective gel-like layer over the intestinal surface epithelium, thereby protecting against bacterial invasion. They are present in both small and the large intestines; however, their number increases, analogous to the expression gradient of mTAS2R131, in the distal parts of the gut, where the concentration of bacteria is the highest [146]. Thus, similar to other extra-oral TAS2Rs, those within colonic goblet cells (Table 2) are speculated to be involved in protecting the gut from enteric pathogens or potentially harmful xenobiotics. Strengthening the defensive role of TAS2Rs in the gut, mTAS2R131 was also identified in a subpopulation of deep-crypt Paneth cells in the ileum (Table 2), which not only produce antimicrobial molecules, but also control stem cell growth and differentiation into all intestinal cell types [147]. However, evidence regarding a direct connection between TAS2R of goblet and Paneth cells and their protective functions is lacking.

Inflammatory bowel disease (IBD) is an umbrella term used to describe disorders involving chronic inflammation of the GI. Its aetiology remains unclear and the pathogenesis is multidimensional and multifactorial, involving interactions among immune, environmental, or genetic factors [148]. TAS2Rs are emerging as regulators of innate immunity in the respiratory tract [77]. Hence, recent studies have suggested that the chemoreceptors in the colon may be directly involved in the modulation of inflammation during IBD via interaction with bacterial metabolites, or via regulation of metabolism. Indirectly, TAS2Rs may determine dietary preferences and intake, which is linked to IBD risk [149]. For example, SNPs within *TAS2R38* (rs713598, rs1726866, and rs10246939) correlated with alcohol and coffee consumption, vegetable and sugar intake, or smoking habits, which are the most ubiquitous environmental factors implicated in IBD [43, 150]. Interestingly, α-gustducin knockout mice were more susceptible to dextran sulphate sodium-induced colitis, with more severe tissue damage and excessive inflammatory responses. The higher levels of TNF and interferon γ and lower levels of IL-10, IL-5, and IL13 in the colon of α-gustducin-null mice illustrated clearly that absence of functional taste-signalling skews immune responses more toward the inflammatory type I immune response than the anti-inflammatory type II response [151]. This may explain the suppressive effects of wild bitter gourd *(Momordica charantia)* fruit extract (BME) on endoplasmic reticulum stress (ER stress), which contributes mostly to the pathogenesis of IBD in LS174T colonic epithelial cells [152–154]. Hence, BME should be considered a potential functional food that can improve intestinal condition by ameliorating ER- and oxidative stress in IBD. However, identification of bitter compounds responsible for ER stress suppression in this and other plants is required, along with a thorough analysis of the downstream mechanisms involved in their action and effects on intestinal cells in vitro, which will assist in verifying their therapeutic potential for IBD in vivo.

#### EECs and enterohormones

##### Ghrelin

Recently, the GI has evolved from being solely a site of nutrient digestion and absorption to the largest endocrine organ in the body, which contains more than 30 genes encoding peptides that act as hormones or other signalling molecules in the gut. An increasing number of studies in both preclinical and clinical settings have also evaluated the effects of TAS2R agonists on enterohormone secretion, albeit with strikingly different outcomes (Tables 1, 2). Of particular importance is ghrelin, the only known peripherally-derived orexigenic molecule, synthesized by the endocrine X/A-like cells of the gastric fundus mucosa. In mice, intragastric gavage of a bitter agonist mixture (DB, PTC, d-(-) salicin, and Q) increased plasma concentrations of octanoylated (*P* < 0.05) and total (*P* < 0.001) ghrelin in a time-dependent manner (without affecting its mRNA expression), suggesting the role of TAS2R stimulation in the release of this hormone. Paradoxically, food intake was only transiently increased during the first 30 min, followed by prolonged and significant reduction over the subsequent 4 h after the administration of bitterants, which is possibly associated with delayed gastric emptying [155]. This endocrine/metabolic effect was not confirmed in another study performed in healthy female volunteers, in whom administration of 10 μmol/kg quinine hydrochloride (QHCl) reduced fasting plasma octanoylated/total ghrelin and motilin levels. Considering the decrease in prospective and actual food intake, along with their covariation with increased activity of homeostatic and hedonic brain regions involved in the control of appetite and food intake, TAS2Rs may represent important alternative neurohumoral gut-brain signals that can mediate the orexigenic effect of ghrelin [156, 157]. Nevertheless, Andreozzi et al. observed that 18 mg QHCl did not affect plasma ghrelin level [158]. This can potentially be explained by insufficiency in the dose of QHCl administered, as well as by the difference in the route of its administration, as enteral application bypasses the stomach, which harbours most X/A-like ghrelin-secreting cells [159]. Thus, further research is required to determine the secretory pattern of ghrelin in response to different bitter ligands and analyse the potential of targeting the gastric TAS2R signalling pathway for preventing obesity.

##### CCK

Mounting evidence of both theoretical and experimental nature indicate that TAS2R-induces secretion of anorexygenic CCK from I-cells (predominantly in the upper small intestine), which elicits various defence responses against food-derived harmful/toxic substances that have bypassed the oral aversive taste-reactivity machinery. Exogenous administration of structurally diverse bitterants (such as DB or PTC) dose-dependently activates TAS2Rs on the STC-1 enteroendocrine cell line, leading to the influx of [Ca^2+^]_i_ via L-type voltage-sensitive Ca^2+^ channels and CCK release [160]. A similar effect was observed in the case of H.g.-12, a steroid glycoside from *Hoodia gordonii*, extract of which exerts appetite-suppressant effect [161]. H.g.-12 is a natural stimulant of CCK secretion from rat ex vivo intestinal preparations, as well as from the human EEC HuTu-80 cell line, which is presumably mediated via hTAS2R14 [162]. This is supported by the observation that in rodents, an intragastric infusion of bitter agonists (DB, PTC, PTU, quinine, and d-(−) salicin) augments plasma CCK and peptide YY (PYY) levels. Subsequently, these act on their receptors (CCK1Rs and Y_2_R) located along the vagal afferents and activate the caudal viscerosensory portion of the nucleus of the solitary tract to condition flavour aversions and delay gastric emptying [163, 164]. Interestingly, according to Joen et al., TAS2R expression in both cultured mouse enteroendocrine cells and mouse intestine is regulated by sterol regulatory element-binding protein 2 (SREBP-2), which is mainly activated by low cellular levels of cholesterol. In addition, TAS2R-dependant CCK secretion was enhanced directly by SREBP-2 in cultured cells and mice fed chow supplemented with lovastatin and ezetimibe to decrease dietary sterol absorption and increase nuclear activity of SREBP-2. A naturally low-cholesterol diet is high in plant material, which is inherently richer in bitter and potentially toxic substances than a high-cholesterol diet, while CCK is well-known for slow absorption rate of lipids and gastric emptying along with food intake. These results suggest that SREBP-2-mediated activation of bitter signalling receptors in the intestine may sensitize the gut to low-fat diet and potential accompanying food-borne toxins that bypass the initial aversive response in the oral cavity [165]. It is also noteworthy that CCK causes gallbladder contraction, releasing its bile acids. One of them, the relatively hydrophilic ursodeoxycholic acid (UDCA) is a known bitter tastant, albeit with unknown receptor [14]. Therefore, UDCA may enhance the SREBP-2-mediated effect, perhaps via positive feedback, by directly acting on intestinal TAS2Rs.

More recently, activation of gut TAS2R, via a CCK signalling machinery was also shown to limit absorption of bitter (and potentially toxic) dietary constituents by up-regulating the transcription of the xenobiotic efflux pump, ABCB1, in both Caco-2 human intestinal cells and mouse intestine [133]. The detoxification was decreased by TAS2R38 siRNA or treatment with YM022, a gastrin receptor antagonist [133]. In addition to the CCK-dependant mechanism, TAS2Rs regulate prostaglandin-dependent intraluminal anion secretion from the large intestine to prevent ingestion of dietary toxins [1, 66].

Apart from targeting detoxification pathways, bitter taste signalling is functionally linked to the regulation of energy intake and appetite control in not only ghrelin-, but also possibly in a CCK-dependant manner. Androzzi et al. [158] have demonstrated that oral administration of 18 mg QHCl in the form of an acid-resistant capsule significantly increased the level of CCK 60 and 90 min after administration of the compound, resulting in reduced calorie intake (ΔKcal) in an ad libitum test (514 ± 248 vs. 569 ± 286 kcal for placebo; *P* = 0.007) in young healthy volunteers. PTC tasters ingested a significantly lower amount of calories when they received QHCl than those who received placebo. Thus, considering the emerging epidemic of over-nutritional diseases and obesity, these observations may be important for their treatment; however, more in-depth experimental and clinical studies are required.

##### Glucagon-like peptide-1

Glucagon-like peptide-1 (GLP-1), an incretin hormone predominantly synthesized postprandially by enteroendocrine L cells (mainly in the distal small and large intestines), mediates intestinal feedback to limit postprandial glycemic excursions and suppresses energy intake [167]. Several cellular studies have visualised a broad spectrum of bitter agonists as GLP-1 secretagogues in taste receptor signalling. For example, the isoquinoline alkaloid, berberine ([C20H18NO4] +), dose-dependently induced secretion of GLP-1 from the mouse STC-1 line [168] and human enteroendocrine NCI-H716 cells via activation of gut-expressed TAS2R38 [169], similar to PTC in the HuTu-80 cell line [170]. These observations were further validated in subsequent studies using reverse transcription-polymerase chain reaction (RT-PCR) along with double-label immunofluorescence assay on small and large intestinal tissues and enteroendocrine cells of both rodents and humans. Silencing of intestinal TAS2Rs by probenecid, or of downstream signalling molecules by their antagonists (Gβγ-gallein; PLCβ2-U73122; IP_3_R_3_-2-APB) substantially attenuated the previously observed incretin hormone secretion in vitro [152, 168, 169, 171–173]. Consistent with these results, a single-dose preload of synthetic or natural bitter compound, its analogue (e.g. KDT501), or a plant extract rich in bitterants has been reported to enhance GLP-1 [139, 152, 172, 173] and insulin [152, 172, 173] concentration, reduce glucose level [152, 172, 173], and subsequently delay gastric emptying in an animal model [155, 174, 175]. Surprisingly, in mice with type 2 diabetes, the blood glucose lowering effect of a 300 mg/kg extract of *Gentiana scabra* containing a bitter iridoid glycoside of loganic acid, was comparable to the effect exerted by 300 mg/kg metformin, a first-line drug for type 2 diabetes mellitus (*P* < 0.01) [173]. Chronic dosing (4-week) of 60 μmol DB/kg/24 h in obese wild type mice caused significant α-gustducin-dependent weight loss and suppression of food intake, accompanied by reduction in orexygenic agouti-related protein or neuropeptide Y transcription and increase in postprandial GLP-1 concentration [176]. Furthermore, Kok et al. demonstrated that a pure analogue of hops-derived isohumulones, KDT501 (in the second phase of clinical trials for the treatment of metabolic disorders), exerts its antidiabetic activity via modulation of bitter taste receptor signalling in the gut, and that GLP-1 is a principal mediator of these effects [139]. However, only few clinical studies in healthy subjects have evaluated the effect of TAS2R signalling on GLP-1 secretion and the associated metabolic effects, while similar studies in patients with obesity and/or type 2 diabetes are lacking. In the human colonic mucosal biopsies, hTAS2R38 colocalised with immunoreactivity for GLP-1, whereas its mRNA expression was similar to those in normal, overweight, or obese people [177]. Importantly, in a cross-over randomised study, Menella et al. observed increase in GLP-1 secretion after breakfast (*P* < 0.05), followed by 30% reduction in energy intake in the post-lunch time period (*P* < 0.04) after oral administration of a microencapsulated bitter ingredient with a core of bitter *Gentiana lutea* root extract and a coating of ethylcellulose-stearate [178]. Intragastric infusion of bitter substances, as mentioned above, demonstrated immediate release of ghrelin, resulting in short-term increase in food intake with subsequent anorexigenic effect starting 4 h after treatment [155]. However, this orexigenic effect was abolished, as the lipid-based bitter ingredient coating was resistant to the hydrolytic environment of the stomach [178]. Thus, the use of microencapsulated bitter compounds that can exert a taste-masking effect can reduce ghrelin secretion. Therefore, over the day, a long-term anorexigenic effect dominates the short-term orexigenic effect, limiting daily energy intake in humans. Taken together, these studies underscore the involvement of TAS2Rs in regulation of energy intake. As the endocannabinoid system (ECS) also participates in controlling feeding behaviour and body composition homeostasis [179], it possibly interact with TAS2Rs. This interpretation is supported by the association between sensitivity to 6-PTU and plasma levels of two endocannabinoids, 2-arachidonylglycerol and arachidonylethanolamide, in normal-weight individuals. Perhaps even more interestingly, TAS2R polymorphisms may influence the feeding behaviour via ECS modulation [180, 181]. Once proven, this hypothesis will undoubtedly encourage further research regarding the causes and mechanism of obesity.

##### PYY

However, unlike other enterohormones, knowledge regarding TAS2R-stimulated PYY secretion by L-type intestinal cells is scarce. Existing data are limited mainly to NCI-H716 enteroendocrine cell lines, in which DB and Q induced PYY release via PLCβ2 in vitro [172]. No study has translated these findings to in vivo animal or human models so far.

#### SCCs

It is noteworthy that a cluster of gustducin-expressing non-endocrine SCCs have also been identified in the mouse stomach [182] and small intestine [183]. In the stomach, they are in close proximity to two subsets of EECs: one population comprises serotonin-secreting enterochromaffin cells, while the other contains the satiety-regulating ghrelin. As the latter are typical “closed-type” EECs that do not contact the stomach lumen and lie against the basal lamina of the surface epithelium, the SCCs may possibly act as sensors for intraluminal contents to regulate ghrelin secretion [182]. This appears highly possible, as DB induced [Ca^2+^]_i_ wave in SCCs expressing TRPM-5, followed by a decrease in [Ca^2+^]_i_ in the adjacent epithelium [140]. Nevertheless, confirmation of TAS2R expression in the gastrointestinal SCCs, apart from those of signalling molecules, is required to verify the above hypothesis.

#### Gastrointestinal smooth muscle cells

Although TAS2Rs are expressed in gastrointestinal smooth muscle cells (Tables 1, 2), their function remains controversial [175]. Indeed, the contractile pattern induced by bitterants was concentration-dependant, region-specific, and agonist-selective. For instance, low concentration of DB (100 μM) induced contraction in mouse fundus, pylorus, and colon, whereas higher concentration (1 mM) unexpectedly relaxed the fundic and pyloric muscle strips [175]. Since shown by Carlson in 1916 [184], bitter substances have been known as inhibitors of gastric emptying, which is consistent with studies conducted many years later in rodents and humans [155, 174, 175, 185, 186]. In wild type mouse, intragastric preload of DB, PTC, or a mixture of bitter substances delayed gastric emptying [155, 175]. Similarly, in healthy volunteers, intragastric infusion of these compounds during the inter-digestive period (fasting) reduced the number of phase III contractions of the migrating motor complex (MMC) [186]. In contrast, the same procedure performed during the postprandial period impaired fundic relaxation and decreased nutrient volume tolerance along with increased satiation in healthy male volunteers [175], or did not affect gastric motility in healthy women [186]. The cause of these discrepancies is unclear at present, but can be due to the effect of age, sex, race, ethnic factors, or geographical area on the TAS2R expression in individual sections of the gastrointestinal tract. So far, both short and long term changes in luminal contents have been shown to alter expression of TAS2Rs and the associated signalling molecules in the mucosa, supporting the proposed role of these structures in luminal chemosensing in the gastrointestinal tract [187, 188]. The molecular mechanism underlying TAS2R-mediated contractility in this area remains an open question, which has to be addressed by conditional loss-of-function experiments in the future. Recent studies have shown that the exposure of the gut to bitter agonists activates the bitter signalling pathway (except TRPM5) and augments [Ca^2+^]_I,_ with further stimulation of calcium-dependent protein kinase and ERK, similar to that observed in pulmonary artery smooth muscle cells [72]. However, in mice harbouring α-gustducin gene knockout, TAS2R-dependent contraction was incompletely attenuated, indicating the involvement of other G-protein subunits expressed in gastrointestinal smooth muscle, such as α-transducin or Gαi. In addition, the contraction amplitude was partially masked by a hyperpolarizing K^+^-efflux induced by bitter agonists. Smooth muscle relaxation, in turn, appears to be not TAS2R-, but PKC-dependent [72, 175]. As the abovementioned studies do not exclude the participation of epithelial TAS2Rs, cooperation between gastrointestinal epithelial and smooth muscle cells can be speculated to be essential for controlling its motor activity. However, experimental studies are required to further confirm this hypothesis. Furthermore, experiments on these receptors as potential therapeutic targets should be conducted to alter gastrointestinal motility and hence interfere with hunger signalling.

### The nervous system

Over the past decades, researchers have detected numerous functional TAS2Rs and their downstream effector proteins, such as α-gustducine, PLCβ2, IP_3_R_3,_ and TRPM5, in animal and human central nervous systems (CNSs), with the highest recorded expression on neuronal populations of the brain stem, hypothalamus, cerebral cortex, cerebellum, nucleus accumbens, hippocampus, and cuboidal epithelial cells of the choroid plexus (CP) located in the ventricular system (Tables 1, 2, 3) [120, 189–193]. Although several exogenous bitter ligands that can cross the blood–brain barrier (BBB), such as food-derived di- and tripeptides [190, 194] or Q [189], have been identified so far, whether these molecules physiologically achieve sufficiently high concentration in the CNS, which is necessary for the activation of brain TAS2Rs, is not known. The importance of these receptors should not be neglected, as some of their bitter agonists show neuroactive [195], neuroprotective [196], or anti-glioma properties [197]. However, the function of the above structures in the nervous system has not yet been determined. As CP performs indispensable functions for the development, maintenance, and functioning of the brain as an active interface between the blood and cerebrospinal fluid (CSF), the taste transduction pathway in the human CP is a likely mechanism for surveying both the blood and CSF, as well as for controlling the traffic of chemical compounds across the BBB [193]. Supporting this, Duarte et al. revealed that TAS2R14 regulates bitter resveratrol transport across the human blood-CSF barrier by modulating the activity of ABC efflux transporters at CP epithelial cells [198]. It is also assumed that TAS2Rs are involved in the secretion of regulatory peptides, such as CCK, responsible for the modulation of food intake and other physiological processes [189]. Furthermore, they are proposed to reactivate endogenous neurogenesis. Indeed, the 72-h incubation of the human SH-SY5Y neuroblastoma (NB) cell line with 100 µM salicin (bitter phenolic glycoside partially penetrating the BBB) stimulated neurite elongation in a TAS2R16-dependent manner with subsequent ERK and cyclic AMP-responsive element binding (CREB) phosphorylation [199]. In addition, overexpression of TAS2R8 and 10 promoted neuronal differentiation in NB cells of the BE(2)C line in vitro. This in turn ameliorated and restored neuronal function. However, further studies are required to understand how TAS2Rs can modulate the proliferative activity of brain stem/progenitor cells in neurogenic niches, their migration to the damaged area, and differentiation into appropriate phenotypes. Explanation of the above may result in development of one of the tools for controlling expansion in situ, which may accelerate the introduction of regenerative therapy in some pathological conditions of CNS, based on stimulation of natural repopulation of damaged neuronal cells.

Analysis of autopsied human brain tissue has shown altered expression of TAS2R mRNA at least in the frontal cortex and substantia nigra of patients with Parkinson's disease, in the frontal and entorhinal cortex of patients with Alzheimer's disease and progressive supranuclear palsy, and in the frontal cortex and cerebellum of patients with Creutzfeldt-Jakob disease [200, 201]. Furthermore, post-mortem studies of brain samples from the dorsolateral prefrontal cortex of patients with chronic schizophrenia revealed down-regulation of hTAS2R4, 5, 13, and 50 mRNA compared to those in control subjects with no history of psychiatric episodes. The expression level of all TAS2Rs was found to correlate inversely with the daily chlorpromazine dose. None of them, however, was associated with the duration of the diseases or the severity of negative symptoms. This suggests that these receptors are possibly involved in the mechanism of action or side effects of antipsychotics [202].

### The cardiovascular system

Expression of both TAS2Rs and downstream signalling pathway in the heart was reported in a series studies by the Foster group using quantitative RT-PCR, in situ hybridization (ISH), and gene-targeted mice reporters (Tables 1, 2, 3) [203–205]. Chromosomal clustering of TAS2R genes mirrored the cardiac expression profile, suggesting that a common gene regulatory block may control the taste receptor locus [203, 204]. In addition, evidence from a study on the Langendorff retrograde-perfused heart model shows that TAS2R activation may exert important negative inotropic effects on cardiac contractility [205]. Notably, the relationship of the SNP rs1376251 in *TAS2R50* with cardiovascular diseases has been also described in numerous prospective population studies, although the underlying mechanism remains unknown [206, 207].

TAS2Rs are expressed on vascular smooth muscle cells of muscles and elastic arteries of human/rodent systemic circulation systems (Tables 1,2,3), and targeting of these receptors induces strong, concentration-dependant, but endothelium-independent vasodilatation in vitro [71, 208] and in vivo [70]. Manson et al. suggested that caveolae, but not Cav1.2 or BKCa channels, are responsible for arterial vasodilator effect [71]. They also demonstrated that bitter agonists, ChQ, dextromethorphan (DXM), and noscapine, lead to relaxation of pre-contracted human pulmonary arteries. Interestingly, contradictory to Manson’s findings (although both may be true), another study demonstrated that DXM, acting via TAS2R1, causes vasoconstrictor responses in the pulmonary circuit and relaxation in the airways [72]. These discrepancies have to be resolved in future.

### The excretory system

mTAS2Rs and α-gustducin are expressed not only in the murine renal corpuscle and tubule [209], including its proximal and distal parts [210], but also in primary renal tubular epithelial cells and the renal collecting tubule M-1 cell line (Table 2) [211]. Importantly, as in the case of the small intestine, mTAS2R expression levels were developmentally regulated in the postnatal period. The first mTAS2Rs were detected as early as 1 week after birth, while significant expression of all receptors (except mTAS2R117) was recorded on the 40th day after birth. As all loops of Henley are short during embryogenesis, in the absence of an ascending thin limb, TAS2Rs may play a critical role in the maturation of the ascending limb of Henley via an unknown mechanism. The function of mTAS2Rs in M1 and primary renal epithelial cells in the presence of bitter coptisine and phelloderine was also demonstrated by assessing downstream calcium influx [211]. Furthermore, using conditional ablation of mTAS2R105^+^ cells to study the renal role of TAS2Rs, increase in the area and diameter of the glomerulus and renal tubule, and decrease in cell density in the glomerulus was identified, which strengthened the pivotal role of local TAS2Rs in maintaining the normal structure of renal tissues [210]. Further supporting this concept, Wooding et al. reported a positive correlation between the hTAS2R43-W35/H212 genotype and Balkan endemic nephropathy (*P* = 0.020; OR = 1.18) [212], a rare cause of progressive atrophy and sclerosis of all structures of the kidney [213]. Intriguingly, although TAS2R43 is the main AA chemodetector, exposure to aristolochic acid (3,4-methylene-dioxoxy-8-methoxy-10-nitrophenanthrene-1-carboxylic acid, AA), a natural alkaloid with well-documented nephrotoxic effects, was believed to be involved in its etiopathogenesis [213].

In addition to preservation of glomerular structure and function, bitter tastants trigger acetylcholine release from polymodal urethral chemosensory cells and induce bladder reflexes, suggesting that TAS2Rs may act as chemodetectors of potential hazardous content in the urethral luminal microenvironment (Tables 1, 2) [214]. Furthermore, activation of these receptors relaxed detrusor smooth muscle and suppressed overactive bladder symptoms in mice with partial bladder outlet obstruction [73].

### The endocrine system

The existence of TAS2Rs and downstream signalling pathways have been recognised in human and mouse thyreocytes, where they probably act as the key players of thyroid hormonogenesis and function (Table 1) [215]. Upon activation of thyreocyte-expressed TAS2Rs, TSH-dependent Ca^2+^ release in Nthy-Ori 3–1 cells decreased in a dose-dependent manner, with simultaneous reduction in iodide efflux. Furthermore, in the Amish, the nonsynonymous coding SNP, rs5020531, located within *hTAS2R42*, correlated with elevated serum levels of free thyroid hormone, especially that of free thyroxin (fT_4_) (*P* = 0.000004). However, the functional consequences of the above modification are unclear, as hTAS2R42 remains an orphan receptor [215].

### Adipose tissue

Recently, TAS2Rs were unexpectedly found to be expressed in the adipose tissue (AT) of both mice and humans, as well as in adipocyte cell lines (Tables 1,2) [176, 216, 217], suggesting their role in regulation of adipogenic patho-/physiological processes. Bitterants have been demonstrated to modulate adipogenesis, although conflicting results exist. Firstly, it was reported that Q robustly induced adipogenesis. Knockdown of mTAS2R106 impaired the pro-adipogenic effect of Q and suppressed the activation of ERK/S6 (extracellular-signal-regulated kinase/Ribosomal protein S6) signaling, one of the main pathway involved in the regulation of adipogenesis [218]. However, in a more recent study, treatment of pre-adipocytes with Q or DB decreased differentiation into mature adipocytes [176]. Moreover, incubation with PROP promoted the delipidation of differentiated adipocytes, indicating that TAS2Rs are also involved in lipid mobilization [219]. The overexpression of TAS2R38 in adipocytes of obese individuals further supports the biological role of TAS2Rs in the AT. This overexpression may be related to the adipocyte hypertrophy that occurs following the expansion of the adipose tissue or represent a new regulatory mechanism [219]. Intriguingly, another independent study showed that TAS2R38 rs10246939 variation was associated with Koreans’ increased obesity risk. Nevertheless the details of underlying mechanism remain elusive, as TAS2R38 rs10246939 genetic variant mostly had a minimal effect on the dietary and energy intake in this Korean cohort study. These findings suggest that other extra-orally expressed TAS2R38 may be associated with energy expenditure-related processes and adiposity metabolism, independent of dietary intake control. For instance, TAS2Rs distributed in thyroid, an important determinant of energy expenditure and basal metabolic rate [220].

### The skin

The presence of TAS2Rs in human skin sections, as well as in keratinocyte cell lines (HaCaT and HPKs), was initially reported in 2014 by Wölfle et al. using immunohistochemical staining, western blotting, and RT-PCR (Table 1). The functionality of TAS2Rs, driving calcium influx in response to stimulation by diphenidol and amarogentin, was also demonstrated in that study [221]. This was supported by results of subsequent studies, which showed that for some TAS2Rs, mRNA abundance was related to presumed sun exposure based on the location from which the skin sample was collected, sex, and age [222, 223]. The activated TAS2R-induced signalling cascades mentioned above have been shown to promote keratinocyte differentiation by stimulating keratin 10, involucrin, and transglutaminase expression [221]. Apart from inducing differentiation, *G. lutea* extract (a rich source of bitter tasting amarogentin), a TAS2R agonist, has been found to enhance lipid synthesis in human keratinocytes, which is essential for building an intact epidermal barrier [224]. Furthermore, amarogentin exerts immunomodulatory effects in the skin by interacting with keratinocytes and mast cells [225]. However, further studies are required to unambiguously demonstrate their TAS2R dependence.

### Common bitter beverages

TAS2R polymorphisms may influence the sensations, liking, or intake of common and nutritionally significant beverages. Among them, several polymorphisms in or near *hTAS2R38*, as well as in *hTAS2R13* and *16*, have been linked to alcohol intake [48, 226–230]. In 2015, Ramos-Lopez et al. identified the novel AVV haplotype for *hTAS2R38;* individuals homozygous for AVV (which increases the bitterness perception threshold) consume significantly more alcoholic beverages than those heterozygous for AVV or PAI homozygotes in the Mexican-Mestizo population [227]. A similar cross-sectional study examining the relationship between hTAS2R43 polymorphism and caffeine consumption supports the idea that this receptor may also be important for liking coffee [231] and perceiving the bitterness of caffeine by humans [232]. However, *hTAS2R43* is not present in the genome of many people [212], indicating that some other bitter signalling mechanism is responsible for perceiving caffeine’s flavour [233]. These can be four SNPs across *hTAS2R3, R4,* and *R5*, forming a haplotype block that explains the variability in taste sensations elicited by coffee, although this association did not affect coffee liking [48].

### Smoking

Although, nicotine-specific TAS2Rs have not yet been identified in humans (contrary to chicken ggTAS2R1) [234], Aoki et al. demonstrated significantly lower expression of several TAS2R genes in smokers vs*.* in non-smokers after adjustments for covariates, such as age and gender [235]. This undoubtedly suggests that the process of smoking cessation may fail owing to well-documented bitter taste impairment among smokers, which is due to tobacco-induced lower-than-normal TAS2R expression [235]. In addition, several studies have investigated the influence of TAS2R38 variants on smoking habits and obtained mixed results [40, 236, 237]. Thus, additional comprehensive analyses are required to verify this association.

### Neoplasms

#### Breast cancer

The distribution of hTAS2R transcripts among human non-cancer breast epithelial cell line (MCF-10A line), as well as in cells derived from estrogen-dependent (MCF-7 line) and estrogen-independent metastatic (MDA-MB-231 line) breast adenocarcinoma, has been investigated, highlighting a strong heterogeneity in hTAS2R expression (Table 4) [238–240]. In particular, the expression of *hTAS2R4* at both mRNA and protein levels, and rapid mobilisation of intracellular calcium as a result of its activation, were significantly lower in MDA-MB-231 and MCF-7 cells than in MCF-10A (*P* < 0.001). This suggested the existence of invasion-metastasis mechanism(s) in which evasion of tumour suppressor genes is modulated by the expression of key genes, such as *hTAS2R4* in breast cancer [238]. In contrast, it can be assumed that TAS2Rs determine the body's physiological defence reaction to metastatic cancer cells, which is supported by the observations of Singh et al. [240]. The authors showed that the activation of TAS2R4 and 14 induced apoptosis, and inhibited cell proliferation and chemotactic migration due to down-regulation of matrix metalloproteinase (MMP)-9 secretion in metastatic breast adenocarcinoma MDA-MB-231.

**Table 4:** mRNA expression level, from highest to lowest, of selected hTAS2R in various cancers and their matched controls

Receptor subtype	Cancer cells and their matched controls	Expression level	P-Value	Refs.
Breast cancer (assessed using qRT-PCR)				
hTAS2R1	Non-cancerous mammary epithelial cell line MCF-10A **(1)** vs Highly metastatic breast cancer cell line MDA-MB-231 **(2)** and poorly metastatic cell line MCF-7 **(3)**	(2) > (1)		[239]
		(3) > (2) > (1)		[238]
hTAS2R3		(2) > (1)		[239]
hTAS2R4		(1) > (2)		[239]
		(1) > (2) > (3)	< 0.001	[238]
hTAS2R5		(1) > (2)		[239]
hTAS2R7		(2) > (1)		[239]
hTAS2R8		(2) > (1)		[239]
hTAS2R9		(1) ~ (2)		[239]
hTAS2R10		(2) > (1)		[239]
		(1) > (2) > (3)		[238]
hTAS2R13		(2) > (1)		[239]
hTAS2R14		(2) > (1)	< 0.001	[239]
hTAS2R16		(1) ~ (1)		[239]
hTAS2R19		(2) > (1)		[239]
hTAS2R20		(2) > (1)	< 0.01	[239]
hTAS2R38		(2) > (1)		[239]
		(3) > (1) > (2)		[238]
hTAS2R39		(2) > (1)		[239]
hTAS2R40		(2) > (1)		[239]
hTAS2R41		(1) > (2)		[239]
hTAS2R42		(1) ~ (2)		[239]
hTAS2R30/31/43/45/46		(2) > (1)	0.001	[239]
hTAS2R49		(2) > (3) > (1)		[238]
hTAS2R50		(2) > (1)		[239]
hTAS2R60		(2) > (1)		[239]
Ovarian cancer (assessed using qRT-PCR)				
hTAS2R1	**A:** Normal fallopian tube tissue **(1)** vs High-grade serous human ovarian cancer cell line OVCAR4 **(2)**, OVCAR8 **(3)** and Ovarian papillary cystadenocarcinoma tissue **(4)** **B:** Normal uterine tissue **(5)** vs Clear cell human ovarian cancer line SKOV3 **(6),** Hypermutated *IGROV1* human ovarian cancer cell line **(7)** and Uterine/endometrial adenocarcinoma cell line HEC1a **(8)**	A: (4) > (1) > (3) > (2) B: (3) > (1) > (2) > (4)	< 0.05 for (3) < 0.05 for (4)	[241]
hTAS2R4		A: (2) > (4) > (1) > (3) B: (1) > (3) > (2) > (4)	< 0.05 for (3) < 0.05 for (2)	[241]
hTAS2R10		A: (2) > (3) > (4) > (1) B: (3) > (4) > (1) > (2)	< 0.01 for (4)	[241]
hTAS2R14		A: (1) > (2) > (3) > (4) B: (1) > (3) > (2) > (4)	< 0.05 for (3); < 0.001 for (4) < 0.01 for (2); < 0.05 for (4)	[241]
hTAS2R38		A: (1) > (2) ~ (3) > (4) B: (1) > (4) > (2) > (3)	< 0.0001 for (2), (3) and (4) < 0.0001 for (2) and (3); < 0.001 for (4)	[241]
Prostate cancer (assessed using qRT-PCR)				
hTAS2R1	Benign prostatic hyperplasia cell line BPH1 **(1)** vs Human prostate cancer cell line PC3 **(2)**, LNCAP **(3)** and DU145 **(4)**	(1) > (2) > (3) > (4)	< 0.05 for (2); < 0.01 for (3); < 0.0001 for (4);	[241]
hTAS2R4		(1) > (3) > (2) > (4)	< 0.001 for (2); < 0.05 for (3); < 0.0001 for (4)	[241]
hTAS2R10		(1) > (4) > (2) > (3)	< 0.01 for (2); < 0.001 for (3)	[241]
hTAS2R14		(1) > (3) > (4) > (2)	< 0.001 for (2) and (4); < 0.01 for (3)	[241]
hTAS2R38		(1) > (2) > (3) > (4)	< 0.05 for (3); < 0.0001 for (4);	[241]
Pancreatic cancer (assessed using qRT-PCR)				
hTAS2R10	Human pancreatic cell line PANC-1 highly resistant to gemcitabine **(1)** vs Human pancreatic cell line BxPC3; more sensitive to gemcitabine **(2)**	(2) > (1)	–	[244]
Neuroblastoma (assessed using qRT-PCR)				
hTAS2R8	More differentiated N-type NB cell line SY5Y **(1)** vs More malignant and less differentiated I-type NB cell line BE(2)C **(2)**	(1) > (2)	< 0.05	[199]
hTAS2R10		(1) > (2)	< 0.05	[199]
Acute myeloid leukaemia (assessed using qRT-PCR)				
hTAS2R1	Primary AML cells **(1)** vs Human leukaemia cell line OCI-AML3 **(2)** and Human leukaemia cell line THP-1 **(3)**	(1) > (2) > (3)	< 0.05	[255]
hTAS2R3		(3) > (2) > (1)	< 0.05	
hTAS2R4		(2) > (1) > (3)	< 0.05	
hTAS2R5		(1) > (3) > (2)	< 0.05	
hTAS2R7		(3) > (1) > (2)	< 0.05	
hTAS2R8		(1) > (2) > (3)	< 0.05	
hTAS2R9		(3) > (1) > (2)	< 0.05	
hTAS2R10		(1) > (3) > (2)	< 0.05	
hTAS2R13		(3) > (1) > (2)	< 0.05	
hTAS2R14		(2) > (3) > (1)	< 0.05	
hTAS2R16		(1)	< 0.05	
hTAS2R20		(3) > (1) > (2)	< 0.05	
hTAS2R30/47		(1) > (3) > (2)	< 0.05	
hTAS2R31		(3) > (2) > (1)	< 0.05	
hTAS2R38		(1) > (3) > (2)	< 0.05	
hTAS2R39		(1) > (2)	< 0.05	
hTAS2R40		(3) > (1)	< 0.05	
hTAS2R41		(1) > (3) > (2)	< 0.05	
hTAS2R43		(1) > (3)	< 0.05	
hTAS2R46		(1) > (3)	< 0.05	
hTAS2R50		(1) > (3) > (2)	< 0.05	

*AML acute myeloma leukaemia, *hTAS2Rs* human bitter taste receptors, *NB* neuroblastoma, *qRT-PCR* quantitative reverse transcription-polymerase chain reaction

#### Ovarian and prostate cancer

Researchers have demonstrated heterologous expression of the receptors in tumours compared to in normal tissue (fallopian tube and uterine tissue; BPH-1 benign prostatic hyperplasia line) in biopsies, along with expression in human ovarian (OVCAR4, OVCAR8, SKOV3 and IGROV1 lines), endometrial (HEC-1a line), and prostate cancer cells (PC3, LNCAP, and DU145 lines) (Table 4) [241]. Low expression of hTAS2R14 and 38 in almost all ovarian cancer lines was accompanied by overexpression of hTAS2R4 and 10, similar to that observed in breast adenocarcinoma cells. In contrast, prostate cancer cells were mostly characterized by reduced expression of the dedicated receptors, except for that of hTAS2R38 in the PC3 line [241]. In addition, 25 μM noscapine (NOS), a bitter isoquinoline alkaloid, induced tumour cell apoptosis in a hTAS2R14-dependent manner, and cell viability after 72 h decreased proportionally with increase in the concentration of the used phytochemical (0–100 μM). Although NOS possesses anti-microtubulin properties similar to paclitaxel, it inhibits the growth of paclitaxel-resistant cancer cells [242], which indicates a difference in the mechanism of action of these two compounds. Therefore, hTAS2R14 appears to be the new target of NOS, which may constitute a new and promising anti-cancer strategy for taxane-resistant cases [241].

#### GI neoplasms

Malignant neoplasms of the GI tract often develop asymptomatically, which results in diagnosis at advanced stages of the disease. In addition, anti-cancer therapy is often ineffective and is associated with various side effects. This "status quo" indicates the need to conduct extensive scientific research that can reveal new and safer approaches for increasing the effectiveness of oncotherapy. The importance of TAS2Rs in pancreatic ductal adenocarcinoma (PDAC) pathogenesis was recognized relatively recently, when Gaida et al. observed cytoplasmic expression of functional TAS2R38 in SU8686, T3M4, and MiaPaCa-2 lines, and in tumour tissues derived from 88 patients. Activation of the above receptor with a bona fide ligand, PTU, or a bacterial quorum detection molecule, AHL-12, led to phosphorylation of p38 mitogen-activated protein (p38MAPK) and ERK1/2 kinases. This was followed by overexpression of the transcription factor—nuclear factor of activated Tcells 1, and the multidrug-resistance protein ABCB1, a transmembrane transporter participating in shuttling a plethora of drugs, such as chemotherapeutics or antibiotics [243]. Another extensive study has systematically investigated the functional role of TAS2R10 expressed on the surface and in the cytoplasm of PDAC tissue and in tumour-derived cell lines (AsPC-1, BxPC-3, Capan-1, COLO-357, MiaPaCa-2, SU.86.86, PANC-1, and T3M4) in the context of chemoresistance (Table 4) [244]. This study demonstrated that caffeine, the natural ligand of this receptor, sensitises cancer cells to two standard chemotherapeutics, gemcitabine and 5-fluorouracil. Knocking down of T2R10 in the BxPC-3 cell line reduced the caffeine-induced effect. Possibly, caffeine triggered Akt phosphorylation and subsequently down-regulated ABCG2 via T2R10, another multi-drug resistance protein that participates in rendering cells resistant to various chemotherapeutics.

The association between TAS2R gene variants and malignant GI tumour susceptibility is currently gaining attention. A study by Choi et al. in the Korean population showed that that the heterozygous *TAS2R38* diplotype (PAV/AVI) significantly increased gastric cancer risk (OR = 1.513; 95% CI = 1.148–1.994) [245]. Similarly, the presence of the AVI/AVI combination of *TAS2R38* was associated with enhanced risk of developing colorectal cancer (CRC) in two different Caucasian populations compared to those with two PAV haplotypes (OR = 1.15; 95% CI = 0.80–1.66 for the Czech population and OR = 1.52; 95% CI = 1.05–2.21 for the German population) [246].

Barontini et al. did not observe any strong influence of the selected SNPs of *TAS2R16* on CRC susceptibility. This is possibly because SNPs in this gene are modulated by lifestyle factor or dietary habit, which were not considered in the cited analysis. However, after stratification by histology (colon vs. rectum), rs1525489 was found to be associated with increased risk of rectal cancer (P_trend_ = 0.0071) [247]. Despite the abundance of results, the role of TAS2R polymorphic variants in carcinogenesis is not clear. On one hand, distinct phenotypes, such as AVI/AVI (non-tester) of *TAS2R38*, reflect differential receptor function and the inability to detect bitter compounds, which may also be a marker for impaired function of the receptors in GI; non-taster individuals eliminate potentially harmful/carcinogenic xenobiotics from the gut slowly and consequently are at high risk of developing GI malignancies [246]. On the other hand, another study has revealed that the AVI/AVI diplotype is not simply a functional marker for the impaired TAS2R38 variant protein, as expression of the homozygous AVI transcript was detected, and subjects with this genotype reacted to other bitter tastes as strongly as individuals with the homozygous PAV variant [248]. This observation may indicate that the structural perturbation of the AVI variant protein may enhance the sensing of other unknown bitter-tasting, potentially carcinogenic molecules, which might initiate appropriate anti-cancer mechanisms [248]. This protective effect of the AVI/AVI diplotype was evident in Choi et al.’s study, in which the AVI haplotype tended to reduce risk of CRC in the Korean population (OR = 0.74; 95% CI = 0.56–0.99) [249]. This may also explain the significantly high diversity observed in TAS2R genes [250]. However, these are only hypotheses, which have to be confirmed using scientific research.

#### Nervous system neoplasms

Khare et al. showed that 6-min exposure of the NG108-15 (mouse NB × rat glioma hybrid) cell line to bitter substances, such as Q, DB, and ranitidine, evoked their intracellular metabolism, which was reflected in the increase in the extracellular acidification rate by 45%, 15%, and 28%, respectively [251]. Thus, it was hypothesized that TAS2Rs in cancer cells play a key role in the development of NB. This was supported by the observations of Seo et al. in human BE(2)C NB cells (Table 4), who showed that up-regulation of endogenous TAS2R8 and 10 suppresses cancer stemness by inducing neuronal cell differentiation, as well as by inhibiting self-renewal capacity and tumorigenicity both in vitro and in vivo. These changes were accompanied by a substantial decrease in migration and invasion potential of NB cells via suppression of the enzymatic activity and expression of MMP-2, and inhibition of the transcriptional activity of hypoxia-inducible factor 1-α and its downstream targets, such as vascular endothelial growth factor and glucose transporter 1 [199]. At the same time, genome-wide association studies have identified the nonsynonymous SNP rs619381 in *TAS2R7*, along with a regulatory SNP rs667128 in *TAS2R8*, which may contribute to glioma risk [252]. The precise pathways and possible physiological functions of TAS2Rs in gliomas, however, remain to be elucidated.

#### Thyroid cancer

Intriguingly, several lines of evidence indicated that Korean females with TAS2R3/4 CC haplotype (rs2270009 and rs2234001, respectively) were at a lower risk of developing papillary thyroid carcinoma than those with the remaining haplotypes (OR = 0.59; 95% CI = 0.36–0.97). Furthermore, total triiodothyronine levels were significantly lower in *TAS2R3/4 CC* haplotype carriers than in other haplotype carriers (*P* = 0.005). Taken together, genetic variations in *TAS2R3/4* may contribute to alteration of the maturation and/or function of thyrocytes and the risk of developing papillary thyroid carcinoma [253]. This concept was partially confirmed by another study showing that triiodothyronine induced the proliferation of papillary thyroid carcinoma cells via up-regulation of cyclin D1 expression [254]. Notably, the role and applicability of TAS2Rs in pillary thyroid carcinoma should be debated.

#### Acute myeloid leucaemia

Salvestrini et al. shed light on the role of TAS2Rs in the extrinsic regulation of leukaemic cell functions [255]. According to them, functional TAS2Rs coupled with the canonical signalling components, such as β-subunit of gustducin and PLC-β2, were detectable in primary leukemic cells of patients with acute myeloid leukaemia (AML) at diagnosis or in human leukaemia cell lines (OCI-AML3, THP-1) (Table 4). Supporting a potential role of these proteins in AML, some TAS2Rs were found to be significantly down-regulated in poor-prognosis AML groups, such as TP53-mut and TET2-mut patients, which might be a strategy adopted by AML cells to evade possible growth-suppression factors, as demonstrated in breast cancer cells [238]. Furthermore, DB treatment altered the expression profile of genes involved in relevant AML cellular processes. Consistent with the results of molecular studies, DB exerted an antiproliferative effect on AML cells by inducing cell cycle arrest in G0/G1 phase or promoted apoptosis via caspase cascade activation. It also attenuated leukaemia cell migration possibly via inhibition of the CXCR4-CXCL12 axis, as well as by inhibiting cellular respiration by decreasing glucose uptake and oxidative phosphorylation. This led to the hypothesis that upon DB exposure, non-proliferating AML cells show reduced cell motility and migration owing to their anchorage in the bone marrow niche, which may protect them from the effects of chemotherapy. It is also noteworthy that TAS2Rs and bitter intrinsic (such as amino acids [14, 20]) or extrinsic (such as drugs [37, 39]) molecules embedded in bone marrow niche might regulate leukaemia cell function; however, further research in this direction is warranted [255].

### Human longevity

Human life expectancy is a complex phenotype affected by a combination of environmental and genetic factors. Genetic factors contribute to human aging by modulating biological pathways, and genetic variation in the TAS2Rs has been shown to affect human longevity [256–258]. It was not until 2012 that Campa and co-workers demonstrated a statistically significant association between rs978739 SNP in *TAS2R16* and longevity (*P* = 0.001) in a Calabria (South Italy) population. In particular, the A/A genotype frequency increases gradually from 35% in subjects aged 20–70 years to up to 55% in centenarians [257]. However, further study on long-lived subjects (LLIs, age range 90–109 years) and young controls (age range 18–45 years) from another area of southern Italy (Cilento) did not confirm this correlation, which could be because of different demographic pressures [258]. Intriguingly, another independent study by Melis et al*.* using the genetically homogenous centenarian cohort (from the central-eastern Sardinia, known as one of the world’s longevity hot spots) showed increased frequency of subjects harbouring the homozygous genotype for the functional variant of *TAS2R38* (PAV/PAV), along with a decreased frequency of those harbouring homozygous genotype for the non-functional form (AVI/AVI), compared to those in the two control cohorts [256]. Nevertheless, the mechanisms underlying these observations still warrant further investigations.

## Limitations and future perspectives

Based on the aforementioned studies, it appears that the perception of TAS2Rs solely as bitterness detectors is outdated and inadequate. The extra-oral expression of these proteins is undoubtedly an intriguing phenomenon that sets new directions for conducting preclinical and clinical research. While planning, the number of restrictions that may hinder or even prevent the evaluation of TAS2R functionality in dedicated tissues and organs of the human body should be considered.

### Application and limitation of methodology for TAS2Rs expression profiling

Standardisation of appropriate methods for identifying TAS2Rs [259, 260] is challenging. The inability to replicate published data is often due to false-positive or false-negative results produced with commercial antibodies that have not been properly validated. The problems are particularly magnified in fields that use antibodies to analyse proteins that are expressed at low levels in cells, as in the case with TAS2Rs [261]. The real solution is proper initial validation of antibodies, which then can provide valuable information regarding the tissue, cellular, and even subcellular location of bitter GPCRs.

However, experts’ view regarding cellular topography of TAS2Rs is strongly divided. By definition, these are membrane receptors, although their intracytoplasmic distribution has been often reported [221, 243, 244]. This finding can be explained by the fact that if most of the cell membrane is covered with the antibody, the staining might appear to be intracellular, although it adheres mostly to the cell surface. Most importantly, TAS2R is subjected to post-translational modifications (including glycosylation and oligomerisation) in the endoplasmic reticulum and Golgi apparatus prior to incorporation into the cell membrane, which might explain its presence in the cytoplasm. Post-translational processing may also itself reduce the availability of TAS2Rs, thereby disrupting the detection and identification of their extracellular and intracellular epitopes using specific antibodies [221, 262]. In addition to the membrane-cytoplasmic distribution, a restricted localisation of TAS2R38 in the nucleus was observed, where it can be involved in some yet unknown functions [263].

As listed in Tables 1, 2, 3, most published scientific reports are based on various techniques used for TAS2R mRNA quantification, including ISH, RT-PCR, or qRT-PCRThe success of ISH for rare mRNAs, such as some extraoral TAS2R molecules, strongly depends on the method of signal amplification revealing low numbers of probe-mRNA hybrids per cell while keeping background staining as low as possible [264]. Technical difficulties of ISH are also evidenced by the large number of different protocols published by researchers [265]. The expression profile of the TAS2Rs is also questionable, as the corresponding genes lack introns [12], and the expression of these proteins in tissues located outside the oral cavity is extremely low, indicating that more amplification cycles are required to increase the sensitivity of the method used [37]. This approach yields satisfactory results, although caution should be exercised when interpreting them owing to the duplication of artefact sequences and associated identification errors.

### Deorphanisation and functional characterization of TAS2Rs

Various techniques have been utilised for identifying TAS2R agonists. However, they have inherent strengths and limitations, and none have all the desired criteria of a suitable assay platform. One major in vitro approach involves the use of an array of heterologous expression systems including, among others, the eukaryotic cell line HEK 293. Using this approach, Lossow et al. identified 80 cognate compounds for 21 mTAS2Rs, 19 of which were previously known orphan receptors [38]. Using the same method, Mayerhof et al. also selected 13 activators for five human orphan receptors, as well as 64 new compounds interacting with previously identified receptors [39]. The main limitation of this type of research is usually low receptor expression or lack of cell surface localisation in heterologous cells [38]. The inability to deorphan more TAS2Rs may also be due to non-functional receptor variants generated by SNPs in the coding region, as was shown by Nelson et al*.* [266]. Here, the authors noted that only two of 24 *TAS2R* genes did not show any amino acid sequence differences when the C57BL/6 and DBA/2 J strains were compared. These changes in the TAS2R sequences may potentially affect ligand response profiles. Finally, the lack or inefficient G protein coupling can interfere with the interpretation of results [267].

Unlike exogenous agonists, knowledge regarding their endogenous counterparts is far from sufficient. The widespread expression of TAS2Rs within the non-gustatory tissues of the organism requires consideration of the existence of intracorporeal, circulating, or locally-produced factors that stimulate these proteins. From among the numerous compounds tested, P4 was the only agonist to stimulate mTAS2R110 and 114, suggesting that this female sex hormone is the endogenous activator of the above structures in mice [38]. However, it is noteworthy that BitterDB lists several other bitter substances naturally found in the human body, which have not been assigned to any receptor so far; in addition to the UDCA mentioned above, they include l-tyrosine and adenosine [14]. Although the interaction between adenosine and mTAS2Rs was initially negated [268], the possibility that the results obtained are influenced by the above-described limiting factors, such as SNP-dependent receptor variability, cannot be excluded. Therefore, these observations constitute an additional incentive for continuing scientific research in this field.

Strikingly, only few known TAS2R antagonists are published. None of these blockers can suppress human bitter taste signalling of all 25 receptors, and considering the ligand diversity of TAS2Rs, it is highly unlikely that a universal blocker can be ever developed. Therefore, it is imperative to develop antagonists with high selectivity and efficacy that can target specific TAS2Rs. A mixture of these blockers can be used to block all the 25 TAS2Rs, if required. Similar to agonists, one antagonist may interact with several receptors, and vice versa, a specific receptor may be blocked by several antagonists [269]. Some of antagonists act as agonists for other TAS2Rs, such as the two sesquiterpene lactones, 3β-hydroxydihydrocostunolide and 3β-hydroxypelenolide [269]. The first small molecule antagonist, 4-(2,2,3-trimethylcyclopentyl)butanoic acid (known as GIV3727), was identified from around 18 000 compounds screened. It possibly acts as an orthostaric insurmountable antagonist, and can also inhibit hTAS2R43, a receptor selective for high-potency non-caloric sweeteners, including saccharine or acesulfame K [270]. The other selective blocker, p-(dipropylsulfamoyl)benzoic acid (better known as probenecid; commonly used as uricosuric agent in the treatment of gout), acts as completely insurmountable antagonist of hTAS2R16 [271]. Similar suppression of bitter taste receptor signalling was observed by naturally occurring flavanones, such as 4′-fluoro-6-methoxyflavanone, 6,3′-dimethoxyflavan, and 6-methoxyflavanone [272]. Low molecular weight compounds are also known as bitterness-masking compounds. Indeed, some umami substances, irrespective of their structure (peptide and non-peptide), can induce receptor-mediated inhibition of bitter ligand binding to human bitter taste receptors [273]. This was further confirmed by Pydi et al., who identified γ-aminobutyric acid (GABA) and Nα,Nα-bis(carboxymethyl)-l-lysine as the first endogenous antagonist and inverse agonist of TAS2R4 [274]. GABA is not only the principal inhibitory neurotransmitter in the adult brain, but is also found outside the CNS, such as is in the airway epithelium, stomach, lungs, kidney, blood cells, pancreas, and testis [275]. This indicates that GABA might play a key role in regulating the function of TAS2R4 in some of these tissues or organs. The antagonist activity of GABA on the remaining 24 TAS2Rs remains to be analysed.

Heterologous expression systems have enabled the deorphanisation and extended characterization of ∼ 60% mouse (21 of 34) [38] and 84% human (21 of 24) [5, 7] TAS2Rs. Most importantly, comparisons of the repertoires of the human and mouse TAS2Rs genes revealed that they may be classified into few main groups [276]. Whereas a one-to-one gene orthology has been observed for some genes of the groups, the others showed patterns of species or lineage-specific duplications. Indeed, the majority of TAS2R genes clustered on human chromosomes 5 and 7 and mouse chromosomes 2 and 15, exhibiting a one-to-one orthology, which indicates that they developed prior to the divergence of primates and rodents, and enabled the recognition of bitter substances that are important to both humans and mice [277]. Theoretically, this offers a valuable window to evaluate the physiological roles of human TAS2Rs in animal models. However, the distinct agonist profiles of sequence-orthologous TAS2Rs presents a particularly challenging problem. One of these receptors is TAS2R38, which recognises its prototypical agonists, 6-n-propylthiouracyl (PROP) and PTC, as bitter in humans, while mice are indifferent to them [278]. This suggests that other mTAS2Rs are responsive to these compounds. Based on this observation, it has been argued that the above receptors mediate detection of species-specific bitter substances, although the existence of additional common bitter agonists for these one-to-one orthologous pairs cannot be excluded [38].

### Regulation of TAS2R expression

The overwhelming scientific research in recent decades has primarily focused on profiling the expression or deorphanisation of TAS2Rs. However, transcriptional and post-transcriptional regulation of their expression is less understood, which, however, will add substantially to knowledge regarding their physiological roles. Several groups have created transgenic mice by incorporating the putative promoter/enhancer region (~ 10-kb fragment upstream of the transcriptional start site of the receptor) for transgene expression specifically in bitter taste receptor-expressing cells of the oral cavity [278, 279]. Regarding the network of extra-oral TAS2Rs, so far only one factor regulating the transcription of these genes has been identified, which was previously known as SREBP-2. It activated the transcription of *TAS2R138* by directly binding to its promoter region in the STC-1 cell line [165]. It is also known that TAS2R sequences within one multigenic cluster reflect a similar expression profile in many different tissues, suggesting a common regulatory mechanism, as confirmed by Foster et al. In their seminal study, areas with putatively high transcriptional activity, identified using histone and DNase I marks, were detected in genome regions in the vicinity of *TAS2R143, 135,* and *126* in the intestine or heart [204]. However, other mechanisms that control the transcription of TAS2R genes might exist, which requires further analysis.

### TAS2Rs and current knowledge in the fields of pharmacology, toxicology, and nutrigenomics

Undeniably, the discovery of extra-oral TAS2Rs has revolutionised the field of pharmacology, toxicology, and nutrigenomics. On one hand, these proteins may be responsible for several side effects associated with the use of various drugs, 30% of which affect GPCRs and most of which possess bitter taste [37, 280]. On the other hand, it is worth debating whether the debiterattion of bitter medications, especially those designed for children, reduces their therapeutic effectiveness. The taste of substances has not been considered in the context of their effects on TAS2Rs. Hence, this aspect should be considered prior to the widespread practice of improving the taste values of food products. In addition, the nature of TAS2R’s response to a specific drug/compound might be SNP-dependent. For example, Nthy-Ori 3-1 cells homozygous for the non-functional *TAS2R38* AVI allele did not respond to PROP [215]. From a practical perspective, future pharmacogenomic studies should explain the relationship between differences in the genome sequence and the patient’s response to the drug for designing personalised medicine.

## Conclusions

The numerous scientific reports concerning extra-oral TAS2Rs appear to be merely the "tip of the bitter iceberg" of hitherto unknown information regarding the extra chemoreceptive role of TAS2Rs in different tissues and organs and the resulting clinical implications. Furthermore, it appears that identification of the network of extra-oral bitter taste receptors is underestimated, as its function is often unclear. The emerging picture shows that genes encoding TAS2Rs may exhibit pleiotropic characteristics and that their protein products are used by various types of cells. Alternatively, they may signal in response to diverse targets that are not related to each other. Bitter sensing probably is one of the functions performed by this cluster of genes, which may play a major role in maintaining the homeostasis of the organism [257]. While unravelling some of the complexities associated with extra-oral TAS2Rs, one can formulate new preventive recommendations and develop personalised pharmacotherapy, thereby overcoming stagnation in the treatment of many diseases, including cancer. A suggestive association between polymorphic genetic variations in these receptors and human longevity provides the required proof-of-concept [256–258]. 

## Abbreviations

[Ca^2^^+^]_i_: Intracellular calcium
6-PTU: 6-Propylthiouracil
β_2_ARs: β2-Adrenoreceptors
ΔKcal: Calorie intake
AA: Kwas arystolochowy
ABC: ATP-binding cassette
ABCB_1_: ATP-binding cassette subfamily B member 1
ABCG_2_: ATP-binding cassette subfamily G member 2
Ach: Acetylcholine
AHL: N-acyl-homoserine lactone
AL: Ascending limb
AML: Acute myeloid leukemia
AMPs: Antimicrobial peptides
ASM: Airway smooth muscle
AT: Adipose tissue
ATP: Adenosine triphosphate
BBB: Blood–brain barrier
BCRP: Breast cancer resistance protein
BitterDB: Database of bitter compounds
BK_Ca_: The large-conductance Ca^2^^+^-activated K + channel
BME: The extract of bitter melon
CALHM1: Calcium homeostasis modulator 1
CALHM3: Calcium homeostasis modulator 3
Ca_v_1.2: Calcium channel, voltage-dependent, L type, alpha 1C subunit
CBF: Ciliary beat frequency
CCCs: Cholinergic chemosensory cells
CCK: Cholecystokinin
CCK_1_Rs: Type 1 CCK receptor
CF: Cystic fibrosis
ChQ: Chloroquine
CNS: Central nervous system
CP: Choroid plexuses
CRC: Collateral cancer
CREB: Cyclic AMP-responsive element binding
CRS: Chronic rhinosinusitis
CSF: Cerebrospinal fluid
CSPs: Competence stimulating peptides
DB: Denatonium benzoate
DXM: Dextromethorphan
ECS: The endocannabinoid system
EECs: Enteroendocrine cells
ER-stress: Endoplasmic reticulum stress
ERK: Extracellular signal-regulated kinases
fTAS2Rs: Feline bitter taste receptors
EVT: Extravillous trophoblast
fT_4_: Free thyroxine
GABA: γ-Aminobutyric acid
ggTAS2Rs: *Gallus gallus* Bitter taste receptors
GI: Gastrointestinal system
GLP-1: Glucagon-like peptide 1
GPCRs: G-protein coupled receptors
GRKs: G-protein-coupled receptor kinases
HSECs: Human sinonasal epithelial cells
hTAS2Rs: Human bitter taste receptors
IBD: Inflammatory bowel disease
ILC2s: Type 2 innate lymphoid cells
IP_3_: Inositol-1,4,5-triphosphate
IP_3_R_3_: Inositol 1,4,5-trisphosphate receptor, type 3
JNK: C-jun N-terminal kinase
LDs: Lipid droplets
LLIs: Long-lived subjects
LPS: Lipopolysaccharide
MΦs: Primary human monocyte-derived unprimed (M0) macrophages
MMC: Migrating motor complex
MMP: Matrix metalloproteinase
MSCs: Mesenchymal stem/stromal cells
mTAS2Rs: Mouse bitter taste receptors
NACh-R: The nicotinic acetylcholine receptor
NB: Neuroblastoma
NF-κB: Nuclear factor kappa-light-chain-enhancer of activated B cells
NK: Natural killer
NK1R: Neurokinin 1 receptor
NO: Nitric oxide
NOS: Noscapine
p38MAPK: P38 mitogen-activated protein kinases
P4: Progesterone
PDAC: Pancreatic ductal adenocarcinoma
PGF2α: Prostaglandin F2 α
P-gp: P-glycoprotein
PI3K: Phosphoinositide 3-kinase
PIBF: Progesterone-induced blocking factor
PLCβ2: 1-Phosphatidylinositol-4,5-bisphosphate phosphodiesterase beta-2
PRAR γ: Peroxisome-proliferator-activated receptor γ
pTAS2Rs: Pig bitter taste receptors
PTC: Phenylthiocarbamide
PTC: Papillary thyroid carcinoma
PYY: Peptide YY
Q: Quinine
QHCl: Quinine hydrochloride
qRT-PCR: Quantitative real-time polymerase chain reaction
QSM: Quorum sensing molecules
ROS: Reactive oxygen species
rTAS2Rs: Rat bitter taste receptors
RT-PCR: Reverse transcription polymerase chain reaction
SCCs: Solitary chemosensory cells
siRNA: Small interfering RNA
SNP: Single nucleotide polymorphism
SOCS3: Suppressor of cytokine signalling 3
SREBP2: Sterol regulatory element-binding protein 2
ST: Syncytiotrophoblast
STAT4: Signal transducer and activator of transcription 4
STAT6: Signal transducer and activator of transcription 6
T1R2/T1R3: Sweet taste receptor
T_3_: Triiodothyronine
TAS2Rs, T2Rs: Bitter taste-sensing type 2 receptors
Tc: The cytotoxic T cells
Tcm: Central memory T cells
Tem: Effector memory T cells
Th: T helper cells
Th1: Type 1 T helper cells
Th2: Type 2 T helper cells
TLR: Toll-like receptor
Tn: Naive T cells
TNF-α: Tumour necrosis factor α
TRCs: Taste-receptor cells
TRPM4: Transient receptor potential cation channel subfamily M member 4
TRPM5: Transient receptor potential cation channel subfamily M member 5
UDCA: Ursodeoxycholic acid
Y_2_R: Neuropeptide Y receptor type 2

## References

[1] Glendinning JI (1994). Is the bitter rejection response always adaptive?. Physiol Behav.

[2] Mishra A, Chaturvedi P, Datta S, Sinukumar S, Joshi P, Garg A (2015). Harmful effects of nicotine. Indian J Med Paediatr Oncol.

[3] Xue AY, Di Pizio A, Levit A (2018). Independent evolution of strychnine recognition by bitter taste receptor subtypes. Front Mol Biosci.

[4] Liman ER, Zhang YV, Montell C (2014). Peripheral coding of taste. Neuron.

[5] Chandrashekar J, Mueller KL, Hoon MA (2000). T2Rs function as bitter taste receptors. Cell.

[6] Chaudhari N, Roper SD (2010). The cell biology of taste. J Cell Biol.

[7] Behrens M, Brockhoff A, Kuhn C, Bufe B, Winnig M, Meyerhof W (2004). The human taste receptor hTAS2R14 responds to a variety of different bitter compounds. Biochem Biophys Res Commun.

[8] Miller IJ. Anatomy of the peripheral taste system. Handb Olfaction Gustation. 1995;521–47.

[9] Höfer D, Püschel B, Drenckhahn D (1996). Taste receptor-like cells in the rat gut identified by expression of alpha-gustducin. Proc Natl Acad Sci U S A.

[10] Wu SV, Rozengurt N, Yang M, Young SH, Sinnett-Smith J, Rozengurt E (2002). Expression of bitter taste receptors of the T2R family in the gastrointestinal tract and enteroendocrine STC-1 cells. Proc Natl Acad Sci U S A.

[11] Finger TE, Böttger B, Hansen A, Anderson KT, Alimohammadi H, Silver WL (2003). Solitary chemoreceptor cells in the nasal cavity serve as sentinels of respiration. Proc Natl Acad Sci U S A.

[12] Lu P, Zhang C-H, Lifshitz LM, ZhuGe R (2017). Extraoral bitter taste receptors in health and disease. J Gen Physiol.

[13] Avau B, Depoortere I (2016). The bitter truth about bitter taste receptors: beyond sensing bitter in the oral cavity. Acta Physiol (Oxf).

[14] Dagan-Wiener A, Di Pizio A, Nissim I (2019). BitterDB: taste ligands and receptors database in 2019. Nucleic Acids Res.

[15] Kazius J, Wurdinger K, van Iterson M, Kok J, Bäck T, IJzerman AP (2008). GPCR NaVa database: natural variants in human G protein-coupled receptors. Hum Mutat.

[16] Roudnitzky N, Behrens M, Engel A (2015). Receptor polymorphism and genomic structure interact to shape bitter taste perception. PLoS Genet.

[17] Adler E, Hoon MA, Mueller KL, Chandrashekar J, Ryba NJP, Zuker CS (2000). A novel family of mammalian taste receptors. Cell.

[18] Nelson G, Hoon MA, Chandrashekar J, Zhang Y, Ryba NJP, Zuker CS (2001). Mammalian sweet taste receptors. Cell.

[19] Behrens M, Foerster S, Staehler F, Raguse J-D, Meyerhof W (2007). Gustatory expression pattern of the human TAS2R bitter receptor gene family reveals a heterogenous population of bitter responsive taste receptor cells. J Neurosci.

[20] Wiener A, Shudler M, Levit A, Niv MY (2012). BitterDB: a database of bitter compounds. Nucleic Acids Res.

[21] Yuan J, Peng G, Xiao G (2020). Xanthohumol suppresses glioblastoma via modulation of Hexokinase 2-mediated glycolysis. J Cancer.

[22] Scagliarini A, Mathey A, Aires V, Delmas D (2020). Xanthohumol, a prenylated flavonoid from hops, induces DNA damages in colorectal cancer cells and sensitizes SW480 cells to the SN38 chemotherapeutic agent. Cells.

[23] Sławińska-Brych A, Zdzisińska B, Czerwonka A (2019). Xanthohumol exhibits anti-myeloma activity in vitro through inhibition of cell proliferation, induction of apoptosis via the ERK and JNK-dependent mechanism, and suppression of sIL-6R and VEGF production. Biochim Biophys Acta (BBA)-Gen Subj..

[24] Engelsgjerd S, Kunnimalaiyaan S, Kandil E, Gamblin TC, Kunnimalaiyaan M (2019). Xanthohumol increases death receptor 5 expression and enhances apoptosis with the TNF-related apoptosis-inducing ligand in neuroblastoma cell lines. PLoS ONE.

[25] Wei S, Sun T, Du J, Zhang B, Xiang D, Li W (2018). Xanthohumol, a prenylated flavonoid from Hops, exerts anticancer effects against gastric cancer in vitro. Oncol Rep.

[26] Carvalho DO, Freitas J, Nogueira P (2018). Xanthohumol inhibits cell proliferation and induces apoptosis in human thyroid cells. Food Chem Toxicol.

[27] Sun Z, Zhou C, Liu F (2018). Inhibition of breast cancer cell survival by Xanthohumol via modulation of the Notch signaling pathway in vivo and in vitro. Oncol Lett.

[28] Saito K, Matsuo Y, Imafuji H (2018). Xanthohumol inhibits angiogenesis by suppressing nuclear factor-κB activation in pancreatic cancer. Cancer Sci.

[29] Pu Q, Guo K, Lin P (2021). Bitter receptor TAS2R138 facilitates lipid droplet degradation in neutrophils during *Pseudomonas aeruginosa* infection. Signal Transduct Target Ther.

[30] Lee RJ, Xiong G, Kofonow JM (2012). T2R38 taste receptor polymorphisms underlie susceptibility to upper respiratory infection. J Clin Invest.

[31] Medapati MR, Singh N, Bhagirath AY (2021). Bitter taste receptor T2R14 detects quorum sensing molecules from cariogenic Streptococcus mutans and mediates innate immune responses in gingival epithelial cells. FASEB J.

[32] Freund JR, Mansfield CJ, Doghramji LJ (2018). Activation of airway epithelial bitter taste receptors by Pseudomonas aeruginosa quinolones modulates calcium, cyclic-AMP, and nitric oxide signaling. J Biol Chem.

[33] Carey RM, Workman AD, Chen B, et al. *Staphylococcus aureus* triggers nitric oxide production in human upper airway epithelium. In: International forum of allergy and rhinology. Wiley Online Library; p. 808–13. 10.1002/alr.21568 PMC4829394 26097237

[34] Carey RM, Chen B, Adappa ND, et al. Human upper airway epithelium produces nitric oxide in response to Staphylococcus epidermidis. In: International forum of allergy and rhinology. Wiley Online Library; p. 1238–44. 10.1002/alr.21837 PMC5903441 27509402

[35] Carey RM, Workman AD, Yan CH (2017). Sinonasal T2R-mediated nitric oxide production in response to Bacillus cereus. Am J Rhinol Allergy.

[36] Verbeurgt C, Veithen A, Carlot S (2017). The human bitter taste receptor T2R38 is broadly tuned for bacterial compounds. PLoS ONE.

[37] Clark AA, Liggett SB, Munger SD (2012). Extraoral bitter taste receptors as mediators of off-target drug effects. FASEB J.

[38] Lossow K, Hübner S, Roudnitzky N (2016). Comprehensive analysis of mouse bitter taste receptors reveals different molecular receptive ranges for orthologous receptors in mice and humans. J Biol Chem.

[39] Meyerhof W, Batram C, Kuhn C (2010). The molecular receptive ranges of human TAS2R bitter taste receptors. Chem Senses.

[40] Risso DS, Kozlitina J, Sainz E (2016). Genetic variation in the TAS2R38 bitter taste receptor and smoking behaviors. PLoS ONE.

[41] Risso DS, Mezzavilla M, Pagani L (2016). Global diversity in the TAS2R38 bitter taste receptor: revisiting a classic evolutionary PROPosal. Sci Rep.

[42] Duffy VB (2007). Variation in oral sensation: implications for diet and health. Curr Opin Gastroenterol.

[43] Dinehart ME, Hayes JE, Bartoshuk LM, Lanier SL, Duffy VB (2006). Bitter taste markers explain variability in vegetable sweetness, bitterness, and intake. Physiol Behav.

[44] Hayes JE, Feeney EL, Nolden AA, McGeary JE (2015). Quinine bitterness and grapefruit liking associate with allelic variants in TAS2R31. Chem Senses.

[45] Allen AL, McGeary JE, Knopik VS, Hayes JE (2013). Bitterness of the non-nutritive sweetener acesulfame potassium varies with polymorphisms in TAS2R9 and TAS2R31. Chem Senses.

[46] Knaapila A, Hwang L-D, Lysenko A (2012). Genetic analysis of chemosensory traits in human twins. Chem Senses.

[47] Reed DR, Zhu G, Breslin PAS (2010). The perception of quinine taste intensity is associated with common genetic variants in a bitter receptor cluster on chromosome 12. Hum Mol Genet.

[48] Hayes JE, Wallace MR, Knopik VS, Herbstman DM, Bartoshuk LM, Duffy VB (2011). Allelic variation in TAS2R bitter receptor genes associates with variation in sensations from and ingestive behaviors toward common bitter beverages in adults. Chem Senses.

[49] Zhang Y, Hoon MA, Chandrashekar J (2003). Coding of sweet, bitter, and umami tastes: different receptor cells sharing similar signaling pathways. Cell.

[50] McLaughlin SK, McKinnon PJ, Margolskee RF (1992). Gustducin is a taste-cell-specific G protein closely related to the transducins. Nature.

[51] Rössler P, Kroner C, Freitag J, Noè J, Breer H (1998). Identification of a phospholipase C β subtype in rat taste cells. Eur J Cell Biol.

[52] Wong GT, Gannon KS, Margolskee RF (1996). Transduction of bitter and sweet taste by gustducin. Nature.

[53] Ruiz CJ, Wray K, Delay E, Margolskee RF, Kinnamon SC (2003). Behavioral evidence for a role of α-gustducin in glutamate taste. Chem Senses.

[54] Dotson CD, Roper SD, Spector AC (2005). PLCβ2-independent behavioral avoidance of prototypical bitter-tasting ligands. Chem Senses.

[55] Pérez CA, Huang L, Rong M (2002). A transient receptor potential channel expressed in taste receptor cells. Nat Neurosci.

[56] Damak S, Rong M, Yasumatsu K (2006). Trpm5 null mice respond to bitter, sweet, and umami compounds. Chem Senses.

[57] Zhang Z, Zhao Z, Margolskee R, Liman E (2007). The transduction channel TRPM5 is gated by intracellular calcium in taste cells. J Neurosci.

[58] Banik DD, Martin LE, Freichel M, Torregrossa A-M, Medler KF (2018). TRPM4 and TRPM5 are both required for normal signaling in taste receptor cells. Proc Natl Acad Sci U S A.

[59] Taruno A, Vingtdeux V, Ohmoto M (2013). CALHM1 ion channel mediates purinergic neurotransmission of sweet, bitter and umami tastes. Nature.

[60] Ma Z, Taruno A, Ohmoto M, et al. CALHM3 is essential for rapid ion channel-mediated purinergic neurotransmission of GPCR-mediated tastes. Neuron. 2018;98(3):547–561. e10. 10.1016/j.neuron.2018.03.043 PMC5934295 29681531

[61] Kinnamon SC (2013). Neurosensory transmission without a synapse: new perspectives on taste signaling. BMC Biol.

[62] Vandenbeuch A, Larson ED, Anderson CB (2015). Postsynaptic P2X3-containing receptors in gustatory nerve fibres mediate responses to all taste qualities in mice. J Physiol.

[63] Bystrova MF, Yatzenko YE, Fedorov IV, Rogachevskaja OA, Kolesnikov SS (2006). P2Y isoforms operative in mouse taste cells. Cell Tissue Res.

[64] Huang YA, Dando R, Roper SD (2009). Autocrine and paracrine roles for ATP and serotonin in mouse taste buds. J Neurosci.

[65] Kataoka S, Toyono T, Seta Y, Ogura T, Toyoshima K (2004). Expression of P2Y 1 receptors in rat taste buds. Histochem Cell Biol.

[66] Deshpande DA, Wang WCH, McIlmoyle EL (2010). Bitter taste receptors on airway smooth muscle bronchodilate by localized calcium signaling and reverse obstruction. Nat Med.

[67] Zhang C-H, Lifshitz LM, Uy KF, Ikebe M, Fogarty KE, ZhuGe R (2013). The cellular and molecular basis of bitter tastant-induced bronchodilation. PLoS Biol.

[68] Grassin-Delyle S, Abrial C, Fayad-Kobeissi S (2013). The expression and relaxant effect of bitter taste receptors in human bronchi. Respir Res.

[69] Tazzeo T, Bates G, Roman HN (2012). Caffeine relaxes smooth muscle through actin depolymerization. Am J Physiol Lung Cell Mol Physiol.

[70] Lund TC, Kobs AJ, Kramer A (2013). Bone marrow stromal and vascular smooth muscle cells have chemosensory capacity via bitter taste receptor expression. PLoS ONE.

[71] Manson ML, Säfholm J, Al-Ameri M (2014). Bitter taste receptor agonists mediate relaxation of human and rodent vascular smooth muscle. Eur J Pharmacol.

[72] Upadhyaya JD, Singh N, Sikarwar AS (2014). Dextromethorphan mediated bitter taste receptor activation in the pulmonary circuit causes vasoconstriction. PLoS ONE.

[73] Zhai K, Yang Z, Zhu X (2016). Activation of bitter taste receptors (tas2rs) relaxes detrusor smooth muscle and suppresses overactive bladder symptoms. Oncotarget.

[74] Parker D, Prince A (2011). Innate immunity in the respiratory epithelium. Am J Respir Cell Mol Biol.

[75] Saunders CJ, Christensen M, Finger TE, Tizzano M (2014). Cholinergic neurotransmission links solitary chemosensory cells to nasal inflammation. Proc Natl Acad Sci U S A.

[76] Barham HP, Cooper SE, Anderson CB, et al. Solitary chemosensory cells and bitter taste receptor signaling in human sinonasal mucosa. In: International forum of allergy and rhinology. Wiley Online Library; p. 450–7. 10.1002/alr.21149 PMC3655139 23404938

[77] Lee RJ, Kofonow JM, Rosen PL (2014). Bitter and sweet taste receptors regulate human upper respiratory innate immunity. J Clin Invest.

[78] Tizzano M, Gulbransen BD, Vandenbeuch A (2010). Nasal chemosensory cells use bitter taste signaling to detect irritants and bacterial signals. Proc Natl Acad Sci U S A.

[79] Hatten KM, Palmer JN, Lee RJ, Adappa ND, Kennedy DW, Cohen NA (2014). Nasal mucus glucose and chronic rhinosinusitis. Otolaryngol Head Neck Surg (1979)..

[80] Garnett JP, Baker EH, Baines DL (2012). Sweet talk: insights into the nature and importance of glucose transport in lung epithelium. Eur Respir J.

[81] Gill SK, Hui K, Farne H (2016). Increased airway glucose increases airway bacterial load in hyperglycaemia. Sci Rep.

[82] Workman AD, Maina IW, Brooks SG (2018). The role of quinine-responsive taste receptor family 2 in airway immune defense and chronic rhinosinusitis. Front Immunol.

[83] Yan CH, Hahn S, McMahon D (2017). Nitric oxide production is stimulated by bitter taste receptors ubiquitously expressed in the sinonasal cavity. Am J Rhinol Allergy.

[84] Adappa ND, Howland TJ, Palmer JN, et al. Genetics of the taste receptor T2R38 correlates with chronic rhinosinusitis necessitating surgical intervention. In: International forum of allergy and rhinology. Wiley Online Library; p. 184–7. 10.1002/alr.21140 23322450

[85] Adappa ND, Zhang Z, Palmer JN, et al. The bitter taste receptor T2R38 is an independent risk factor for chronic rhinosinusitis requiring sinus surgery. In: International forum of allergy and rhinology. Wiley Online Library; p. 3–7. 10.1002/alr.21253 PMC4082560 24302675

[86] Mfuna Endam L, Filali‐Mouhim A, Boisvert P, Boulet L, Bossé Y, Desrosiers M. Genetic variations in taste receptors are associated with chronic rhinosinusitis: a replication study. In: International forum of allergy and rhinology. Wiley Online Library; p. 200–6. 10.1002/alr.21275 24415641

[87] Adappa ND, Farquhar D, Palmer JN, et al. TAS2R38 genotype predicts surgical outcome in nonpolypoid chronic rhinosinusitis. In: International forum of allergy and rhinology. Wiley Online Library; p. 25–33. 10.1002/alr.21666 PMC4830631 26562612

[88] Adappa ND, Workman AD, Hadjiliadis D, et al. T2R38 genotype is correlated with sinonasal quality of life in homozygous ΔF508 cystic fibrosis patients. In: International forum of allergy & rhinology. Wiley Online Library; p. 356–61. 10.1002/alr.21675 PMC4830383 26678226

[89] Kim U, Jorgenson E, Coon H, Leppert M, Risch N, Drayna D (2003). Positional cloning of the human quantitative trait locus underlying taste sensitivity to phenylthiocarbamide. Science (80-)..

[90] Gallo S, Grossi S, Montrasio G (2016). TAS2R38 taste receptor gene and chronic rhinosinusitis: new data from an Italian population. BMC Med Genet.

[91] Karunasagar A, Garag SS, Appannavar SB, Kulkarni RD, Naik AS (2018). Bacterial biofilms in chronic rhinosinusitis and their implications for clinical management. Indian J Otolaryngol Head Neck Surg.

[92] Cantone E, Negri R, Roscetto E (2018). In vivo biofilm formation, gram-negative infections and TAS 2 R 38 polymorphisms in CRS w NP patients. Laryngoscope.

[93] Adappa ND, Truesdale CM, Workman AD, et al. Correlation of T2R38 taste phenotype and in vitro biofilm formation from nonpolypoid chronic rhinosinusitis patients. In: International forum of allergy and rhinology. Wiley Online Library; p. 783–91. 10.1002/alr.21803 PMC5500301 27309535

[94] Safi C, Zheng Z, Dimango E, Keating C, Gudis DA (2019). Chronic rhinosinusitis in cystic fibrosis: diagnosis and medical management. Med Sci.

[95] Castaldo A, Cernera G, Iacotucci P (2020). TAS2R38 is a novel modifier gene in patients with cystic fibrosis. Sci Rep.

[96] Shah AS, Ben-Shahar Y, Moninger TO, Kline JN, Welsh MJ (2009). Motile cilia of human airway epithelia are chemosensory. Science (80-)..

[97] Krasteva G, Canning BJ, Hartmann P (2011). Cholinergic chemosensory cells in the trachea regulate breathing. Proc Natl Acad Sci U S A.

[98] Pulkkinen V, Manson ML, Säfholm J, Adner M, Dahlén S-E (2012). The bitter taste receptor (TAS2R) agonists denatonium and chloroquine display distinct patterns of relaxation of the guinea pig trachea. Am J Physiol Lung Cell Mol Physiol.

[99] Deshpande DA, Robinett KS, Wang WCH, Sham JSK, An SS, Liggett SB (2011). Bronchodilator activity of bitter tastants in human tissue. Nat Med.

[100] Robinett KS, Koziol-White CJ, Akoluk A, An SS, Panettieri RA, Liggett SB (2014). Bitter taste receptor function in asthmatic and nonasthmatic human airway smooth muscle cells. Am J Respir Cell Mol Biol.

[101] An SS, Wang WCH, Koziol-White CJ (2012). TAS2R activation promotes airway smooth muscle relaxation despite β2-adrenergic receptor tachyphylaxis. Am J Physiol Lung Cell Mol Physiol.

[102] Israel E, Drazen JM, Liggett SB (2000). The effect of polymorphisms of the β2-adrenergic receptor on the response to regular use of albuterol in asthma. Am J Respir Crit Care Med.

[103] Yoon S-Y, Shin E-S, Park SY (2016). Association between polymorphisms in bitter taste receptor genes and clinical features in Korean asthmatics. Respiration.

[104] Tan X, Sanderson MJ (2014). Bitter tasting compounds dilate airways by inhibiting airway smooth muscle calcium oscillations and calcium sensitivity. Br J Pharmacol.

[105] Shifren A, Witt C, Christie C, Castro M (2012). Mechanisms of remodeling in asthmatic airways. J Allergy.

[106] Nayak AP, Deshpande DA, Penn RB (2018). New targets for resolution of airway remodeling in obstructive lung diseases. F1000Research..

[107] Sharma P, Panebra A, Pera T (2016). Antimitogenic effect of bitter taste receptor agonists on airway smooth muscle cells. Am J Physiol Lung Cell Mol Physiol.

[108] Kim D, Cho S, Castaño MA, Panettieri RA, Woo JA, Liggett SB (2019). Biased TAS2R bronchodilators inhibit airway smooth muscle growth by downregulating phosphorylated extracellular signal-regulated kinase 1/2. Am J Respir Cell Mol Biol.

[109] Pan S, Sharma P, Shah SD, Deshpande DA (2017). Bitter taste receptor agonists alter mitochondrial function and induce autophagy in airway smooth muscle cells. Am J Physiol Lung Cell Mol Physiol.

[110] Orsmark-Pietras C, James A, Konradsen JR (2013). Transcriptome analysis reveals upregulation of bitter taste receptors in severe asthmatics. Eur Respir J.

[111] Ekoff M, Choi J-H, James A, Dahlén B, Nilsson G, Dahlén S-E (2014). Bitter taste receptor (TAS2R) agonists inhibit IgE-dependent mast cell activation. J Allergy Clin Immunol.

[112] Grassin Delyle S, Abrial C, Brollo M, Fayad-Kobeissi S, Naline E, Devillier P. Characterization of the expression and the role of bitter taste receptors in human lung parenchyma and macrophages. In: D36 sepsis, acute respiratory distress syndrome, and acute lung injury. American Thoracic Society; 2014. p. A5749–A5749.

[113] Malki A, Fiedler J, Fricke K, Ballweg I, Pfaffl MW, Krautwurst D (2015). Class I odorant receptors, TAS1R and TAS2R taste receptors, are markers for subpopulations of circulating leukocytes. J Leukoc Biol.

[114] Maurer S, Wabnitz GH, Kahle NA (2015). Tasting Pseudomonas aeruginosa biofilms: human neutrophils express the bitter receptor T2R38 as sensor for the quorum sensing molecule N-(3-oxododecanoyl)-l-homoserine lactone. Front Immunol.

[115] Gaida MM, Dapunt U, Hänsch GM (2016). Sensing developing biofilms: the bitter receptor T2R38 on myeloid cells. Pathog Dis.

[116] Tran HTT, Herz C, Ruf P, Stetter R, Lamy E (2018). Human T2R38 bitter taste receptor expression in resting and activated lymphocytes. Front Immunol.

[117] Gopallawa I, Freund JR, Lee RJ. Bitter taste receptors stimulate phagocytosis in human macrophages through calcium, nitric oxide, and cyclic-GMP signaling. Cell Mol Life Sci. 2020;1–16. 10.1007/s00018-020-03494-y PMC7492447 32172302

[118] Guerrouahen BS, Sidahmed H, Al Sulaiti A, Al Khulaifi M, Cugno C (2019). Enhancing mesenchymal stromal cell immunomodulation for treating conditions influenced by the immune system. Stem Cells Int..

[119] Pinti M, Appay V, Campisi J (2016). Aging of the immune system: focus on inflammation and vaccination. Eur J Immunol.

[120] Voigt A, Hübner S, Lossow K, Hermans-Borgmeyer I, Boehm U, Meyerhof W (2012). Genetic labeling of Tas1r1 and Tas2r131 taste receptor cells in mice. Chem Senses.

[121] Panneck AR, Rafiq A, Schütz B (2014). Cholinergic epithelial cell with chemosensory traits in murine thymic medulla. Cell Tissue Res.

[122] Soultanova A, Voigt A, Chubanov V (2015). Cholinergic chemosensory cells of the thymic medulla express the bitter receptor Tas2r131. Int Immunopharmacol.

[123] Zhang Y, Wang X, Li X (2017). Identification of a specific agonist of human TAS2R14 from Radix Bupleuri through virtual screening, functional evaluation and binding studies. Sci Rep.

[124] Grassin-Delyle S, Salvator H, Mantov N (2019). Bitter taste receptors (TAS2Rs) in human lung macrophages: receptor expression and inhibitory effects of TAS2R agonists. Front Physiol.

[125] Wölfle U, Elsholz FA, Kersten A, Haarhaus B, Schumacher U, Schempp CM (2016). Expression and functional activity of the human bitter taste receptor TAS2R38 in human placental tissues and JEG-3 cells. Molecules.

[126] Taher S, Borja Y, Cabanela L (2019). Cholecystokinin, gastrin, cholecystokinin/gastrin receptors, and bitter taste receptor TAS2R14: trophoblast expression and signaling. Am J Physiol Regul Integr Comp Physiol.

[127] Warning JC, McCracken SA, Morris JM (2011). A balancing act: mechanisms by which the fetus avoids rejection by the maternal immune system. Reproduction.

[128] Larsen B, Hwang J (2011). Progesterone interactions with the cervix: translational implications for term and preterm birth. Infect Dis Obstet Gynecol.

[129] Shah N (2016). The in vitro interrogation of the immune system in pregnancy.

[130] Klaffenbach D, Friedrich D, Strick R (2011). Contribution of different placental cells to the expression and stimulation of antimicrobial proteins (AMPs). Placenta.

[131] Dietrich CG, Geier A, Elferink RPJO (2003). ABC of oral bioavailability: transporters as gatekeepers in the gut. Gut.

[132] Han LW, Gao C, Mao Q (2018). An update on expression and function of P-gp/ABCB1 and BCRP/ABCG2 in the placenta and fetus. Expert Opin Drug Metab Toxicol.

[133] Jeon T-I, Seo Y-K, Osborne TF (2011). Gut bitter taste receptor signalling induces ABCB1 through a mechanism involving CCK. Biochem J.

[134] Zheng K, Lu P, Delpapa E (2017). Bitter taste receptors as targets for tocolytics in preterm labor therapy. FASEB J.

[135] Xu J, Cao J, Iguchi N, Riethmacher D, Huang L (2012). Functional characterization of bitter-taste receptors expressed in mammalian testis. Mol Hum Reprod.

[136] Li F, Zhou M (2012). Depletion of bitter taste transduction leads to massive spermatid loss in transgenic mice. Mol Hum Reprod.

[137] Governini L, Semplici B, Pavone V (2020). Expression of taste receptor 2 subtypes in human testis and sperm. J Clin Med.

[138] Gentiluomo M, Crifasi L, Luddi A (2017). Taste receptor polymorphisms and male infertility. Hum Reprod.

[139] Kok BP, Galmozzi A, Littlejohn NK (2018). Intestinal bitter taste receptor activation alters hormone secretion and imparts metabolic benefits. Mol Metab.

[140] Bezençon C, Fürholz A, Raymond F (2008). Murine intestinal cells expressing Trpm5 are mostly brush cells and express markers of neuronal and inflammatory cells. J Comp Neurol.

[141] Clavenzani P, De Giorgio R, Mazzoni M (2009). Expression of alpha-transducin, a chemoreceptive molecule, in endocrine and non-endocrine cells of the pig gastrointestinal tract. Vet Res Commun.

[142] Luo X-C, Chen Z-H, Xue J-B (2019). Infection by the parasitic helminth *Trichinella spiralis* activates a Tas2r-mediated signaling pathway in intestinal tuft cells. Proc Natl Acad Sci.

[143] Gerbe F, Sidot E, Smyth DJ (2016). Intestinal epithelial tuft cells initiate type 2 mucosal immunity to helminth parasites. Nature.

[144] Howitt MR, Lavoie S, Michaud M (2016). Tuft cells, taste-chemosensory cells, orchestrate parasite type 2 immunity in the gut. Science (80-)..

[145] von Moltke J, Ji M, Liang H-E, Locksley RM (2016). Tuft-cell-derived IL-25 regulates an intestinal ILC2–epithelial response circuit. Nature.

[146] Prandi S, Bromke M, Hübner S (2013). A subset of mouse colonic goblet cells expresses the bitter taste receptor Tas2r131. PLoS ONE.

[147] Prandi S, Voigt A, Meyerhof W, Behrens M (2018). Expression profiling of Tas2r genes reveals a complex pattern along the mouse GI tract and the presence of Tas2r131 in a subset of intestinal Paneth cells. Cell Mol Life Sci.

[148] Kim DH, Cheon JH (2017). Pathogenesis of inflammatory bowel disease and recent advances in biologic therapies. Immune Netw.

[149] Turner A, Chijoff E, Veysey M (2019). Interactions between taste receptors and the gastrointestinal microbiome in inflammatory bowel disease. J Nutr Intermed Metab..

[150] Chamoun E, Mutch DM, Allen-Vercoe E (2018). A review of the associations between single nucleotide polymorphisms in taste receptors, eating behaviors, and health. Crit Rev Food Sci Nutr.

[151] Feng P, Chai J, Yi H (2018). Aggravated gut inflammation in mice lacking the taste signaling protein α-gustducin. Brain Behav Immun.

[152] Huang T, Lu K-N, Pai Y-P, Hsu C, Huang C (2013). Role of GLP-1 in the hypoglycemic effects of wild bitter gourd. Evid-Based Complement Altern Med..

[153] Cao SS (2015). Endoplasmic reticulum stress and unfolded protein response in inflammatory bowel disease. Inflamm Bowel Dis.

[154] Kunde DA, Chong WC, Nerurkar PV (2017). Bitter melon protects against ER stress in LS174T colonic epithelial cells. BMC Complement Altern Med.

[155] Janssen S, Laermans J, Verhulst P-J, Thijs T, Tack J, Depoortere I (2011). Bitter taste receptors and α-gustducin regulate the secretion of ghrelin with functional effects on food intake and gastric emptying. Proc Natl Acad Sci U S A.

[156] Deloose E, Corsetti M, Van Oudenhove L, Depoortere I, Tack J (2018). Intragastric infusion of the bitter tastant quinine suppresses hormone release and antral motility during the fasting state in healthy female volunteers. Neurogastroenterol Motil.

[157] Iven J, Biesiekierski JR, Zhao D (2019). Intragastric quinine administration decreases hedonic eating in healthy women through peptide-mediated gut-brain signaling mechanisms. Nutr Neurosci.

[158] Andreozzi P, Sarnelli G, Pesce M (2015). The bitter taste receptor agonist quinine reduces calorie intake and increases the postprandial release of cholecystokinin in healthy subjects. J Neurogastroenterol Motil.

[159] Peeters TL (2005). Ghrelin: a new player in the control of gastrointestinal functions. Gut.

[160] Chen MC, Wu SV, Reeve JR, Rozengurt E (2006). Bitter stimuli induce Ca2+ signaling and CCK release in enteroendocrine STC-1 cells: role of L-type voltage-sensitive Ca2+ channels. Am J Physiol Cell Physiol.

[161] Zhang S, Ma Y, Li J, Ma J, Yu B, Xie X (2014). Molecular matchmaking between the popular weight-loss herb *Hoodia gordonii* and GPR119, a potential drug target for metabolic disorder. Proc Natl Acad Sci U S A.

[162] Le Nevé B, Foltz M, Daniel H, Gouka R (2010). The steroid glycoside Hg-12 from *Hoodia gordonii* activates the human bitter receptor TAS2R14 and induces CCK release from HuTu-80 cells. Am J Physiol Gastrointest Liver Physiol.

[163] Hao S, Dulake M, Espero E, Sternini C, Raybould HE, Rinaman L (2009). Central Fos expression and conditioned flavor avoidance in rats following intragastric administration of bitter taste receptor ligands. Am J Physiol Regul Integr Comp Physiol.

[164] Hao S, Sternini C, Raybould HE (2008). Role of CCK1 and Y2 receptors in activation of hindbrain neurons induced by intragastric administration of bitter taste receptor ligands. Am J Physiol Regul Integr Comp Physiol.

[165] Jeon T-I, Zhu B, Larson JL, Osborne TF (2008). SREBP-2 regulates gut peptide secretion through intestinal bitter taste receptor signaling in mice. J Clin Invest.

[166] Kaji I, Karaki S, Fukami Y, Terasaki M, Kuwahara A (2009). Secretory effects of a luminal bitter tastant and expressions of bitter taste receptors, T2Rs, in the human and rat large intestine. Am J Physiol Gastrointest Liver Physiol.

[167] Donnelly D (2012). The structure and function of the glucagon-like peptide-1 receptor and its ligands. Br J Pharmacol.

[168] Yue X, Liang J, Gu F, Du D, Chen F (2018). Berberine activates bitter taste responses of enteroendocrine STC-1 cells. Mol Cell Biochem.

[169] Yu Y, Hao G, Zhang Q (2015). Berberine induces GLP-1 secretion through activation of bitter taste receptor pathways. Biochem Pharmacol.

[170] Pham H, Hui H, Morvaridi S (2016). A bitter pill for type 2 diabetes? The activation of bitter taste receptor TAS2R38 can stimulate GLP-1 release from enteroendocrine L-cells. Biochem Biophys Res Commun.

[171] Dotson CD, Zhang L, Xu H (2008). Bitter taste receptors influence glucose homeostasis. PLoS ONE.

[172] Kim K-S, Egan JM, Jang H-J (2014). Denatonium induces secretion of glucagon-like peptide-1 through activation of bitter taste receptor pathways. Diabetologia.

[173] Suh H-W, Lee K-B, Kim K-S (2015). A bitter herbal medicine *Gentiana scabra* root extract stimulates glucagon-like peptide-1 secretion and regulates blood glucose in db/db mouse. J Ethnopharmacol.

[174] Glendinning JI, Yiin Y-M, Ackroff K, Sclafani A (2008). Intragastric infusion of denatonium conditions flavor aversions and delays gastric emptying in rodents. Physiol Behav.

[175] Avau B, Rotondo A, Thijs T (2015). Targeting extra-oral bitter taste receptors modulates gastrointestinal motility with effects on satiation. Sci Rep.

[176] Avau B, Bauters D, Steensels S (2015). The gustatory signaling pathway and bitter taste receptors affect the development of obesity and adipocyte metabolism in mice. PLoS ONE.

[177] Latorre R, Huynh J, Mazzoni M (2016). Expression of the bitter taste receptor, T2R38, in enteroendocrine cells of the colonic mucosa of overweight/obese vs lean subjects. PLoS ONE.

[178] Mennella I, Fogliano V, Ferracane R, Arlorio M, Pattarino F, Vitaglione P (2016). Microencapsulated bitter compounds (from *Gentiana lutea*) reduce daily energy intakes in humans. Br J Nutr.

[179] Silvestri C, Di Marzo V (2013). The endocannabinoid system in energy homeostasis and the etiopathology of metabolic disorders. Cell Metab.

[180] Barbarossa IT, Carta G, Murru E (2013). Taste sensitivity to 6-n-propylthiouracil is associated with endocannabinoid plasma levels in normal-weight individuals. Nutrition.

[181] Tepper BJ, Banni S, Melis M, Crnjar R, Tomassini BI (2014). Genetic sensitivity to the bitter taste of 6-n-propylthiouracil (PROP) and its association with physiological mechanisms controlling body mass index (BMI). Nutrients.

[182] Hass N, Schwarzenbacher K, Breer H (2007). A cluster of gustducin-expressing cells in the mouse stomach associated with two distinct populations of enteroendocrine cells. Histochem Cell Biol.

[183] Sutherland K, Young RL, Cooper NJ, Horowitz M, Blackshaw LA (2007). Phenotypic characterization of taste cells of the mouse small intestine. Am J Physiol Gastrointest Liver Physiol.

[184] Carlson AJ (1917). The control of hunger in health and disease. AJN Am J Nurs.

[185] Andrews CN, Verschueren S, Janssen P (2013). Su2072 the bitter taste receptor agonist denatonium benzoate alters intragastric pressure profiles during nutrient drink test in healthy volunteers. Gastroenterology.

[186] Deloose E, Janssen P, Corsetti M (2017). Intragastric infusion of denatonium benzoate attenuates interdigestive gastric motility and hunger scores in healthy female volunteers. Am J Clin Nutr.

[187] Mazzoni M, De Giorgio R, Latorre R (2013). Expression and regulation of α-transducin in the pig gastrointestinal tract. J Cell Mol Med.

[188] Vegezzi G, Anselmi L, Huynh J (2014). Diet-induced regulation of bitter taste receptor subtypes in the mouse gastrointestinal tract. PLoS ONE.

[189] Singh N, Vrontakis M, Parkinson F, Chelikani P (2011). Functional bitter taste receptors are expressed in brain cells. Biochem Biophys Res Commun.

[190] Dehkordi O, Rose JE, Fatemi M (2012). Neuronal expression of bitter taste receptors and downstream signaling molecules in the rat brainstem. Brain Res.

[191] Tomás J, Santos CRA, Quintela T, Gonçalves I (2016). “Tasting” the cerebrospinal fluid: another function of the choroid plexus?. Neuroscience.

[192] Chao DHM, Argmann C, Van Eijk M (2016). Impact of obesity on taste receptor expression in extra-oral tissues: emphasis on hypothalamus and brainstem. Sci Rep.

[193] Duarte AC, Santos J, Costa AR (2020). Bitter taste receptors profiling in the human blood-cerebrospinal fluid-barrier. Biochem Pharmacol.

[194] Upadhyaya J, Pydi SP, Singh N, Aluko RE, Chelikani P (2010). Bitter taste receptor T2R1 is activated by dipeptides and tripeptides. Biochem Biophys Res Commun.

[195] Li Y, Yu L, Zhao L, Zeng F, Liu Q (2017). Resveratrol modulates cocaine-induced inhibitory synaptic plasticity in VTA dopamine neurons by inhibiting phosphodiesterases (PDEs). Sci Rep.

[196] Raygude KS, Kandhare AD, Ghosh P, Bodhankar SL (2012). Anticonvulsant effect of fisetin by modulation of endogenous biomarkers. Biomed Prev Nutr.

[197] Jhaveri N, Cho H, Torres S (2011). Noscapine inhibits tumor growth in TMZ-resistant gliomas. Cancer Lett.

[198] Duarte AC, Rosado T, Costa AR (2020). The bitter taste receptor TAS2R14 regulates resveratrol transport across the human blood-cerebrospinal fluid barrier. Biochem Pharmacol.

[199] Seo Y, Kim Y-S, Lee KE, Park TH, Kim Y (2017). Anti-cancer stemness and anti-invasive activity of bitter taste receptors, TAS2R8 and TAS2R10, in human neuroblastoma cells. PLoS ONE.

[200] Ansoleaga B, Garcia-Esparcia P, Llorens F, Moreno J, Aso E, Ferrer I (2013). Dysregulation of brain olfactory and taste receptors in AD, PSP and CJD, and AD-related model. Neuroscience.

[201] Garcia-Esparcia P, Schlüter A, Carmona M (2013). Functional genomics reveals dysregulation of cortical olfactory receptors in Parkinson disease: novel putative chemoreceptors in the human brain. J Neuropathol Exp Neurol.

[202] Ansoleaga B, Garcia-Esparcia P, Pinacho R, Haro JM, Ramos B, Ferrer I (2015). Decrease in olfactory and taste receptor expression in the dorsolateral prefrontal cortex in chronic schizophrenia. J Psychiatr Res.

[203] Foster SR, Porrello ER, Purdue B (2013). Expression, regulation and putative nutrient-sensing function of taste GPCRs in the heart. PLoS ONE.

[204] Foster SR, Porrello ER, Stefani M (2015). Cardiac gene expression data and in silico analysis provide novel insights into human and mouse taste receptor gene regulation. Naunyn-Schmiedebergs Arch Pharmacol.

[205] Foster SR, Blank K, See Hoe LE (2014). Bitter taste receptor agonists elicit G-protein-dependent negative inotropy in the murine heart. FASEB J.

[206] Shiffman D, O’Meara ES, Bare LA (2008). Association of gene variants with incident myocardial infarction in the Cardiovascular Health Study. Arterioscler Thromb Vasc Biol.

[207] Akao H, Polisecki E, Kajinami K (2012). KIF6, LPA, TAS2R50, and VAMP8 genetic variation, low density lipoprotein cholesterol lowering response to pravastatin, and heart disease risk reduction in the elderly. Atherosclerosis.

[208] Chen J-G, Ping N-N, Liang D (2017). The expression of bitter taste receptors in mesenteric, cerebral and omental arteries. Life Sci.

[209] Rajkumar P, Aisenberg WH, Acres OW, Protzko RJ, Pluznick JL (2014). Identification and characterization of novel renal sensory receptors. PLoS ONE.

[210] Liu X, Gu F, Jiang L, Chen F, Li F (2015). Expression of bitter taste receptor Tas2r105 in mouse kidney. Biochem Biophys Res Commun.

[211] Liang J, Chen F, Gu F, Liu X, Li F, Du D (2017). Expression and functional activity of bitter taste receptors in primary renal tubular epithelial cells and M-1 cells. Mol Cell Biochem.

[212] Wooding SP, Atanasova S, Gunn HC, Staneva R, Dimova I, Toncheva D (2012). Association of a bitter taste receptor mutation with Balkan Endemic Nephropathy (BEN). BMC Med Genet.

[213] Jelaković B, Dika Ž, Arlt VM, et al. Balkan endemic nephropathy and the causative role of aristolochic acid. In: Seminars in nephrology. Elsevier; p. 284–96. 10.1016/j.semnephrol.2019.02.007 31054628

[214] Deckmann K, Filipski K, Krasteva-Christ G (2014). Bitter triggers acetylcholine release from polymodal urethral chemosensory cells and bladder reflexes. Proc Natl Acad Sci.

[215] Clark AA, Dotson CD, Elson AET (2015). TAS2R bitter taste receptors regulate thyroid function. FASEB J.

[216] Kimura S, Kato E (2020). TAS2R expression profile in brown adipose, white adipose, skeletal muscle, small intestine, liver and common cell lines derived from mice. Gene Rep..

[217] Alexandre J, Malheiro R, Dias da Silva D, Carmo H, Carvalho F, Silva JP (2020). The synthetic cannabinoids THJ-2201 and 5F-PB22 enhance in vitro CB1 receptor-mediated neuronal differentiation at biologically relevant concentrations. Int J Mol Sci.

[218] Ning X, He J, Shi X, Yang G (2016). Regulation of adipogenesis by quinine through the ERK/S6 pathway. Int J Mol Sci.

[219] Cancello R, Micheletto G, Meta D (2020). Expanding the role of bitter taste receptor in extra oral tissues: TAS2R38 is expressed in human adipocytes. Adipocyte.

[220] Choi J-H (2019). Variation in the TAS2R38 bitterness receptor gene was associated with food consumption and obesity risk in Koreans. Nutrients.

[221] Wölfle U, Elsholz FA, Kersten A, Haarhaus B, Müller WE, Schempp CM (2015). Expression and functional activity of the bitter taste receptors TAS2R1 and TAS2R38 in human keratinocytes. Skin Pharmacol Physiol.

[222] Reszka E, Nowakowska-Świrta E, Kupczyk M (2015). Expression of bitter taste receptors in the human skin in vitro. J Clin Res Bioeth.

[223] Shaw L, Mansfield C, Colquitt L (2018). Personalized expression of bitter ‘taste’receptors in human skin. PLoS ONE.

[224] Wölfle U, Haarhaus B, Seiwerth J (2017). The herbal bitter drug Gentiana lutea modulates lipid synthesis in human keratinocytes in vitro and in vivo. Int J Mol Sci.

[225] Wölfle U, Haarhaus B, Schempp CM (2015). Amarogentin displays immunomodulatory effects in human mast cells and keratinocytes. Mediat Inflamm..

[226] Wang JC, Hinrichs AL, Bertelsen S (2007). Functional variants in TAS2R38 and TAS2R16 influence alcohol consumption in high-risk families of African-American origin. Alcohol Clin Exp Res.

[227] Ramos-Lopez O, Roman S, Martinez-Lopez E (2015). Association of a novel TAS2R38 haplotype with alcohol intake among Mexican-Mestizo population. Ann Hepatol.

[228] Dotson CD, Wallace MR, Bartoshuk LM, Logan HL (2012). Variation in the gene TAS2R13 is associated with differences in alcohol consumption in patients with head and neck cancer. Chem Senses.

[229] Duffy VB, Davidson AC, Kidd JR (2004). Bitter receptor gene (TAS2R38), 6-n-propylthiouracil (PROP) bitterness and alcohol intake. Alcohol Clin Exp Res.

[230] Fu D, Riordan S, Kieran S, Andrews RA, Ring HZ, Ring BZ (2019). Complex relationship between TAS2 receptor variations, bitterness perception, and alcohol consumption observed in a population of wine consumers. Food Funct.

[231] Pirastu N, Kooyman M, Traglia M (2014). Association analysis of bitter receptor genes in five isolated populations identifies a significant correlation between TAS2R43 variants and coffee liking. PLoS ONE.

[232] Ledda M, Kutalik Z, Souza Destito MC (2014). GWAS of human bitter taste perception identifies new loci and reveals additional complexity of bitter taste genetics. Hum Mol Genet.

[233] Poole RL, Tordoff MG (2017). The taste of caffeine. J Caffeine Res.

[234] Lipchock SV, Spielman AI, Mennella JA (2017). Caffeine bitterness is related to daily caffeine intake and bitter receptor mRNA abundance in human taste tissue. Perception.

[235] Aoki M, Takao T, Takao K, Koike F, Suganuma N (2014). Lower expressions of the human bitter taste receptor TAS2R in smokers: reverse transcriptase-polymerase chain reaction analysis. Tob Induc Dis.

[236] Mangold JE, Payne TJ, Ma JZ, Chen G, Li MD (2008). Bitter taste receptor gene polymorphisms are an important factor in the development of nicotine dependence in African Americans. J Med Genet.

[237] Cannon DS, Baker TB, Piper ME (2005). Associations between phenylthiocarbamide gene polymorphisms and cigarette smoking. Nicotine Tob Res.

[238] Singh N, Chakraborty R, Bhullar RP, Chelikani P (2014). Differential expression of bitter taste receptors in non-cancerous breast epithelial and breast cancer cells. Biochem Biophys Res Commun.

[239] Jaggupilli A, Singh N, Upadhyaya J (2017). Analysis of the expression of human bitter taste receptors in extraoral tissues. Mol Cell Biochem.

[240] Singh N, Shaik FA, Myal Y, Chelikani P (2020). Chemosensory bitter taste receptors T2R4 and T2R14 activation attenuates proliferation and migration of breast cancer cells. Mol Cell Biochem.

[241] Martin LTP, Nachtigal MW, Selman T (2019). Bitter taste receptors are expressed in human epithelial ovarian and prostate cancers cells and noscapine stimulation impacts cell survival. Mol Cell Biochem.

[242] Zhou J, Gupta K, Yao J (2002). Paclitaxel-resistant human ovarian cancer cells undergo c-Jun NH2-terminal kinase-mediated apoptosis in response to noscapine. J Biol Chem.

[243] Gaida MM, Mayer C, Dapunt U (2016). Expression of the bitter receptor T2R38 in pancreatic cancer: localization in lipid droplets and activation by a bacteria-derived quorum-sensing molecule. Oncotarget.

[244] Stern L, Giese N, Hackert T (2018). Overcoming chemoresistance in pancreatic cancer cells: role of the bitter taste receptor T2R10. J Cancer.

[245] Choi J-H, Lee J, Choi IJ, Kim Y-W, Ryu KW, Kim J (2016). Genetic variation in the TAS2R38 bitter taste receptor and gastric cancer risk in Koreans. Sci Rep.

[246] Carrai M, Steinke V, Vodicka P (2011). Association between TAS2R38 gene polymorphisms and colorectal cancer risk: a case-control study in two independent populations of Caucasian origin. PLoS ONE.

[247] Barontini J, Antinucci M, Tofanelli S (2017). Association between polymorphisms of TAS2R16 and susceptibility to colorectal cancer. BMC Gastroenterol.

[248] Bufe B, Breslin PAS, Kuhn C (2005). The molecular basis of individual differences in phenylthiocarbamide and propylthiouracil bitterness perception. Curr Biol.

[249] Choi J-H, Lee J, Oh JH (2017). Variations in the bitterness perception-related genes TAS2R38 and CA6 modify the risk for colorectal cancer in Koreans. Oncotarget.

[250] Kim U, Wooding S, Ricci D, Jorde LB, Drayna D (2005). Worldwide haplotype diversity and coding sequence variation at human bitter taste receptor loci. Hum Mutat.

[251] Khare S, Gokulan K, Linthicum DS (2001). Cellular responses of NG108-15 and SK-N-MC lines to sweet and bitter tastants as measured by extracellular acidification rates. J Neurosci Res.

[252] Lee YH, Song GG (2015). Genome-wide pathway analysis in glioma. Neoplasma.

[253] Choi J-H, Lee J, Yang S, Lee EK, Hwangbo Y, Kim J (2018). Genetic variations in TAS 2 R3 and TAS 2 R4 bitterness receptors modify papillary carcinoma risk and thyroid function in Korean females. Sci Rep.

[254] Perri A, Catalano S, Bonofiglio D (2014). T3 enhances thyroid cancer cell proliferation through TRβ1/Oct-1-mediated cyclin D1 activation. Mol Cell Endocrinol.

[255] Salvestrini V, Ciciarello M, Pensato V (2020). Denatonium as a bitter taste receptor agonist modifies transcriptomic profile and functions of acute myeloid leukemia cells. Front Oncol.

[256] Melis M, Errigo A, Crnjar R, Pes GM, Barbarossa IT (2019). TAS2R38 bitter taste receptor and attainment of exceptional longevity. Sci Rep.

[257] Campa D, De Rango F, Carrai M (2012). Bitter taste receptor polymorphisms and human aging. PLoS ONE.

[258] Malovini A, Accardi G, Aiello A (2019). Taste receptors, innate immunity and longevity: the case of TAS2R16 gene. Immun Ageing.

[259] Jensen BC, Swigart PM, Simpson PC (2009). Ten commercial antibodies for alpha-1-adrenergic receptor subtypes are nonspecific. Naunyn Schmiedebergs Arch Pharmacol.

[260] Herrera M, Sparks MA, Alfonso-Pecchio AR, Harrison-Bernard LM, Coffman TM (2013). Lack of specificity of commercial antibodies leads to misidentification of angiotensin type 1 receptor protein. Hypertension.

[261] Schonbrunn A (2014). Antibody can get it right: confronting problems of antibody specificity and irreproducibility.

[262] Behrens M, Born S, Redel U (2012). Immunohistochemical detection of TAS2R38 protein in human taste cells. PLoS ONE.

[263] Dżaman K, Zagor M, Sarnowska E, Krzeski A, Kantor I (2016). The correlation of TAS2R38 gene variants with higher risk for chronic rhinosinusitis in Polish patients. Otolaryngol Pol Polish Otolaryngol.

[264] Yang H, Wanner IB, Roper SD, Chaudhari N (1999). An optimized method for in situ hybridization with signal amplification that allows the detection of rare mRNAs. J Histochem Cytochem.

[265] Jensen E (2014). Technical review: in situ hybridization. Anat Rec.

[266] Nelson TM, Munger SD, Boughter JD (2005). Haplotypes at the Tas2r locus on distal chromosome 6 vary with quinine taste sensitivity in inbred mice. BMC Genet.

[267] Ozeck M, Brust P, Xu H, Servant G (2004). Receptors for bitter, sweet and umami taste couple to inhibitory G protein signaling pathways. Eur J Pharmacol.

[268] Dando R, Dvoryanchikov G, Pereira E, Chaudhari N, Roper SD (2012). Adenosine enhances sweet taste through A2B receptors in the taste bud. J Neurosci.

[269] Brockhoff A, Behrens M, Roudnitzky N, Appendino G, Avonto C, Meyerhof W (2011). Receptor agonism and antagonism of dietary bitter compounds. J Neurosci.

[270] Slack JP, Brockhoff A, Batram C (2010). Modulation of bitter taste perception by a small molecule hTAS2R antagonist. Curr Biol.

[271] Greene TA, Alarcon S, Thomas A (2011). Probenecid inhibits the human bitter taste receptor TAS2R16 and suppresses bitter perception of salicin. PLoS ONE.

[272] Roland WSU, Gouka RJ, Gruppen H (2014). 6-Methoxyflavanones as bitter taste receptor blockers for hTAS2R39. PLoS ONE.

[273] Rhyu M-R, Kim Y, Misaka T (2020). Suppression of hTAS2R16 signaling by umami substances. Int J Mol Sci.

[274] Pydi SP, Sobotkiewicz T, Billakanti R, Bhullar RP, Loewen MC, Chelikani P (2014). Amino acid derivatives as bitter taste receptor (T2R) blockers. J Biol Chem.

[275] Erdö SL, Wolff JR (1990). γ-Aminobutyric acid outside the mammalian brain. J Neurochem.

[276] Conte C, Ebeling M, Marcuz A, Nef P, Andres-Barquin PJ (2002). Identification and characterization of human taste receptor genes belonging to the TAS2R family. Cytogenet Genome Res.

[277] Shi P, Zhang J, Yang H, Zhang Y (2003). Adaptive diversification of bitter taste receptor genes in Mammalian evolution. Mol Biol Evol.

[278] Mueller KL, Hoon MA, Erlenbach I, Chandrashekar J, Zuker CS, Ryba NJP (2005). The receptors and coding logic for bitter taste. Nature.

[279] Ohmoto M, Maeda N, Abe K, Yoshihara Y, Matsumoto I (2010). Genetic tracing of the neural pathway for bitter taste in t2r5-WGA transgenic mice. Biochem Biophys Res Commun.

[280] Rask-Andersen M, Masuram S, Schiöth HB (2014). The druggable genome: evaluation of drug targets in clinical trials suggests major shifts in molecular class and indication. Annu Rev Pharmacol Toxicol.

