# Correction to: An update on extra-oral bitter taste receptors

**DOI:** 10.1186/s12967-021-03137-1

**Published:** 2021-11-26

**Authors:** Kamila Tuzim, Agnieszka Korolczuk

**Affiliations:** Department of Clinical Pathomorphology, Medical University of Lublin, ul. Jaczewskiego 8b, 20-090 Lublin, Poland

## Correction to: J Transl Med (2021) 19:440 10.1186/s12967-021-03067-y

After publication of the original article [1] the authors noticed the following error:

- The author names were listed as “Tuzim Kamila and Korolczuk Agnieszka”.
  - The correct author names are Kamila Tuzim and Agnieszka Korolczuk.

Furthermore, several minor typos’ have been corrected. The original article has been updated.
